# Automatic identification of medically important mosquitoes using embedded learning approach-based image-retrieval system

**DOI:** 10.1038/s41598-023-37574-3

**Published:** 2023-06-30

**Authors:** Veerayuth Kittichai, Morakot Kaewthamasorn, Yudthana Samung, Rangsan Jomtarak, Kaung Myat Naing, Teerawat Tongloy, Santhad Chuwongin, Siridech Boonsang

**Affiliations:** 1 Faculty of Medicine, King Mongkut’s Institute of Technology Ladkrabang, Bangkok, Thailand; 2 Veterinary Parasitology Research Unit, Faculty of Veterinary Science, Chulalongkorn University, Bangkok, Thailand; 3 Faculty of Tropical Medicine, Mahidol University, Bangkok, Thailand; 4 Faculty of Science and Technology, Suan Dusit University, Bangkok, Thailand; 5 College of Advanced Manufacturing Innovation, King Mongkut’s Institute of Technology Ladkrabang, Bangkok, Thailand; 6 Department of Electrical Engineering, School of Engineering, King Mongkut’s Institute of Technology Ladkrabang, Bangkok, Thailand

**Keywords:** Computational biology and bioinformatics, Mathematics and computing

## Abstract

Mosquito-borne diseases such as dengue fever and malaria are the top 10 leading causes of death in low-income countries. Control measure for the mosquito population plays an essential role in the fight against the disease. Currently, several intervention strategies; chemical-, biological-, mechanical- and environmental methods remain under development and need further improvement in their effectiveness. Although, a conventional entomological surveillance, required a microscope and taxonomic key for identification by professionals, is a key strategy to evaluate the population growth of these mosquitoes, these techniques are tedious, time-consuming, labor-intensive, and reliant on skillful and well-trained personnel. Here, we proposed an automatic screening, namely the deep metric learning approach and its inference under the image-retrieval process with Euclidean distance-based similarity. We aimed to develop the optimized model to find suitable miners and suggested the robustness of the proposed model by evaluating it with unseen data under a 20-returned image system. During the model development, well-trained ResNet34 are outstanding and no performance difference when comparing five data miners that showed up to 98% in its precision even after testing the model with both image sources: stereomicroscope and mobile phone cameras. The robustness of the proposed—trained model was tested with secondary unseen data which showed different environmental factors such as lighting, image scales, background colors and zoom levels. Nevertheless, our proposed neural network still has great performance with greater than 95% for sensitivity and precision, respectively. Also, the area under the ROC curve given the learning system seems to be practical and empirical with its value greater than 0.960. The results of the study may be used by public health authorities to locate mosquito vectors nearby. If used in the field, our research tool in particular is believed to accurately represent a real-world scenario.

## Introduction

Mosquito-borne diseases such as dengue fever, zika, and malaria are a public health concern, which currently being top 10 leading causes of death in low-income countries, partly due to a healthcare service disruption during the COVID-19 outbreak^1^. These diseases are prevalent in tropical and subtropical areas due to human mobility: globalization, labor movement, public transport, and climate changes. The disease transmission and spread throughout the region are associated with the factorized population density and blood-feeding and seeking behaviors of mosquito vectors^2^. Disease prevention-based automatic device has been encouraged to develop and deploy to control strategy in the entomological field^3^. A conventional microscopic identification with a consult of taxonomic key is accepted as a gold standard^4^, however, it is time-consuming, labor-intensive and needs highly skilled and trained personnel. Effectively surveillance based on a simple and reliable identification method for mosquito species is required for further control strategy.

Conventional observation under a stereomicroscope, the intact mosquito identification-based taxonomy key under stereomicroscope taxonomy by skilled and experienced entomologists. This technique is normally used to distinguish the intact mosquito species by determining various external characteristics; including (1) proboscis and palpi based color and pattern of the head, (2) body integument and abdomen, (3) wing patterns, (4) mesonotum of thorax and (5) femur and tarsi of legs^5,6^. Several studies are relied on the conventional observation with the intact mosquito identification-based taxonomy key by skilled and experienced entomologists^7,8^. The method mentioned is prone to errors caused by humans and external factors such as variation of specimens from distinct geographical range^8^. Unfortunately, un-intact mosquito samples may be found during the field-caught and/ or preservation processes^9,10^. These damaged- and incomplete morphological characteristics and decolorization are consequently causing a lack of key elements necessary for species identification by using the standard taxonomic key. This is because the deformity of the morphological features of the mosquitoes limits the precision of its classification^11^. As the problem proposed as above lead us to acquire a need for an effective method to identification with accuracy and challenging with wide range species of the mosquito vectors to guide the effective intervention strategy. Although high throughput techniques (such as PCR, Real-Time PCR, and DNA barcoding) have been used to replace conventional procedures^12–14^, the following techniques suffer from many drawbacks including the leisurely-speed of the detection and the lack of qualified molecular biologists. Alternatively, a rapid approach to enhancing the species identification of the mosquito vector is needed.

Automatic/ computerized assisting tools for species classification of the mosquito vector have been intensively studied^9,11,15,16^. Advancing computerized devices can be represented by several major classification methods such as artificial intelligence (AI), machine learning (ML), and deep learning (DL) based convolutional neural networks (CNNs). Previously, Rustam et al.^17^ reported that using a Machine Learning (ETC model) and Deep Learning (VGG16) facilitate to classify two medically important mosquitoes, *Aedes* and *Culex *species. Those species are mainly distributed in tropical area.

Several versions of ML with the dimensionality reduction have been widely studied. For instance, gene expression data obtained from malaria mosquito vector dataset can be managed with popular feature extraction such as independent component analysis (ICA)^18,19^, hybrid techniques between principal component analysis (PCA) and ICA^20^ and ANOVA-ant colony optimization approach^21^. The extracted feature was classified by machine learning such as support vector machine (SVM), k nearest neighbors (KNN) and Decision Tree classifiers. As a result, the technique showed an improved classification accuracy and cost effective to find the relationship among relevant genes, which can be useful for clinicians in decision making. CNN-classification was successfully conducted and developed by using several types of input such as image characteristics and a wing-beat for insects^22–24^. Previous studies proposed that vector population density could be determined by mosquito data^25–28^; including the eggs and the body and wings which have been investigated to classify cryptic species of malaria mosquito^4,29^. The wing-morphometric method, however, experiences several barriers; it is time-consuming to require skills and expertise while preparing and mounting wing samples onto slide-glasses. Hence, the use of the whole body for the characteristic-feature analysis is preferably as it is the closet condition and the most realistic object, with no special equipment for processing needed.

Since an object classification problem may be affected by class imbalance and data scarcity, deep metric learning (DML)-based semantic distance of data points including the content-based image retrieval (CBIR) analyses could give a better alternative. Previous studies have achieved to implement the DML and dimensional reduction to classify the medical insect as effective^30,31^. A malaria mosquito species, namely *Anopheles arabiensis*, was observed by using Mid-infrared spectroscopy (MIRS) as dataset that was learnt by dimensionality reduction and transfer learning techniques. The study showed high accuracy to ~ 98% accuracy for predicting mosquito age classes, representing dynamic population of the mosquito vector specific to a region^30^. In addition, Merchan and colleges (2023) introduced the use of Deep Metric Learning models based two neural network backbones; Siamese neural networks (SNNs) and Triplet neural networks (TNNs), to classify malaria mosquito species and tick species. High performance of the trained model obtained that showed upto 99% and 93% accuracy for identify Culicidae and Ixodidae’s families, respectively^31^. The CBIR demonstrates the use of a query image on a train database^32–35^. The technique relies on the functions of embedding losses to embed feature-vectors onto a space^36^. Although slow convergence from a large proportion of data triplet-wise is encountered with pairwise or triplet-wise losses, the Pytorch Triplet-Margin loss was shown to be the proper alternative one in DML works^37,38^. Main component of the CBIR is the image’s embedding as such transformation of images from Euclidean to multidimensional representatives, lower-dimensional manifolds, giving potential retrieval systems more accurate and faster^35,39,40^. The feature labels and model optimization seem to be a key component for image-retrieval tasks. Nowadays, DML technique is widely used in medical research^39–41^. The clinical applications of CBIR were potentially assisted technicians by observing content-based pathological images and leading diagnosis by searching referent specimens from the compiling database^42–46^. The CBIR also reduced an inefficient way for clinicians to spend a lot of time seeking textbook/ taxonomic key/ guidance for confirmed tasks. Popularly-CBIR application was used to deal with multi-sources of chest X-ray image for the COVID-19 pandemic^35^. Previously, CBIR implementation for classifying several histological data yielded recall at 84.04% in top-1 recommendation (Recall@1)^34,47^. The interpretation of digital images provided a timely diagnosis depending on the image retrieval system, specifically, for both physician/ radiologist examination and computer-aided diagnosis (CAD). Conceptually, the diagnosis of the similar/ambiguous images can provide a most probable answer for the queried image. Although traditional-DML has been studied for the medical area, no such development of the image retrieval system has been reported in the entomological field.

As several achievements studied using DML reported as above, it interests us to implement it to our work. This is because the model used aims to extract and to learn object features as multidimensional features vectors (representing the distance and locations), assigned in a neighboring space that have similar features due to the distances between them are minimized^48^. Considering DL attempts to define characteristics of each object’s class based on percentage of probability, nevertheless, DML learns to measure the similarities between object within any class by generating an embedding in in low-dimensional space where similar features of any object locate closer. Here may be the concept learning to support excellent result obtained and give the result better beyond deep learning approaches. The aims of the present study were to develop a simple and user-friendly automatic identification tool for medically important mosquitoes. We generated: (i) two datasets; (1) captured by using a stereomicroscope and (2) the other one used a microscope within a mobile phone. (ii) trained-model comparison for identifying the gender and also species of field-caught mosquito vectors was investigated whether the trained model with stereomicroscopic images to classify the test set from mobile phone images and vice versa. (iii) the combination of both different-image sources was trained and tested by using unknown slitted from the same sources depending on k-neighbors neighbors for 20-image retrieval. All model developments were trained based on the Resnet-34 as a neural network backbone and the embedding feature vector relied on the triplet-margin Loss as feature-vector embedding function. The findings of the study could aid public health personnel in identifying mosquito vectors in the surrounding area. In particular, the tool from our research is thought to reflect an actual scenario if used in the field.

## Materials and methods

### Ethics statement

This research design was approved by the Animal Research Ethics Committee, King Mongkut’s Institute of Technology Ladkrabang with ACUC-KMITL-RES/2021/003 (This is a condition of Thailand research fund regulations). This study was carried out in accordance with ARRIVE guidelines (https://arriveguidelines.org).

### Mosquito datasets

In the study, archived mosquito species identified by expert entomologists were used^16,49,50^. Images were photographed by using two-independent equipment including a camera-adhere mobile phone and a Nikon SMZ745 microscope mounted to a Nikon DS series digital camera (Table 1). Two-different datasets were constructed in order to train a deep neural network model to come forward to a realistic situation when applying the model-embedded mobile phone application. A non-mosquito species, namely *Musca domestica*, was included in the study which was used to confirm that the trained-model could distinguish the mosquito out from non-mosquito.

**Table 1:** Sample size of image set used.

No	Insect species	Index	Mobile phone			Stereomicroscope			Combination		
			Total	Train	Test	Total	Train	Test	Total	Train	Test
1	Non-mosquito (*Musca domestica*)	mNM	254	229	25	–	–	–	254	229	25
2	*Anopheles dirus*, female	Adir_f	87	78	9	123	111	12	210	189	21
3	*Anopheles dirus*, male	Adir_m	857	771	86	151	136	15	1008	907	101
4	*Aedes aegypti*, female	Aeg_f	175	157	18	141	127	14	316	284	32
5	*Aedes aegypti*, male	Aeg_m	151	136	15	263	237	26	414	373	41
6	*Aedes albopictus*, female	Alb_f	1131	1018	113	314	283	31	1445	1301	144
7	*Aedes albopictus*, male	Alb_m	563	507	56	101	91	10	664	598	66
8	*Armigeres subalbatus*, female	Asub_f	617	555	62	283	255	28	900	810	90
9	*Culex quinquefasciatus*, female	Cqui_f	316	284	32	119	107	12	435	391	44
10	*Culex quinquefasciatus*, male	Cqui_m	558	502	56	154	139	15	712	641	71
11	*Culex gelidus*, female	Cgel_f	–	–	–	341	307	34	341	307	34
12	*Culex vishnui*, female	Cvis_f	–	–	–	229	206	23	229	206	23
13	*Mansonia annularis*, female	Mann_f	–	–	–	150	135	15	150	135	15
14	*Mansonia Indiana*, female	Mind_f	–	–	–	438	394	44	438	394	44
15	*Mansonia uniformis*, female	Muni_f	–	–	–	166	149	17	166	149	17
Total = 7682			4709	4237	472	2973	2677	296	7682	6914	768

**Figure 1:**
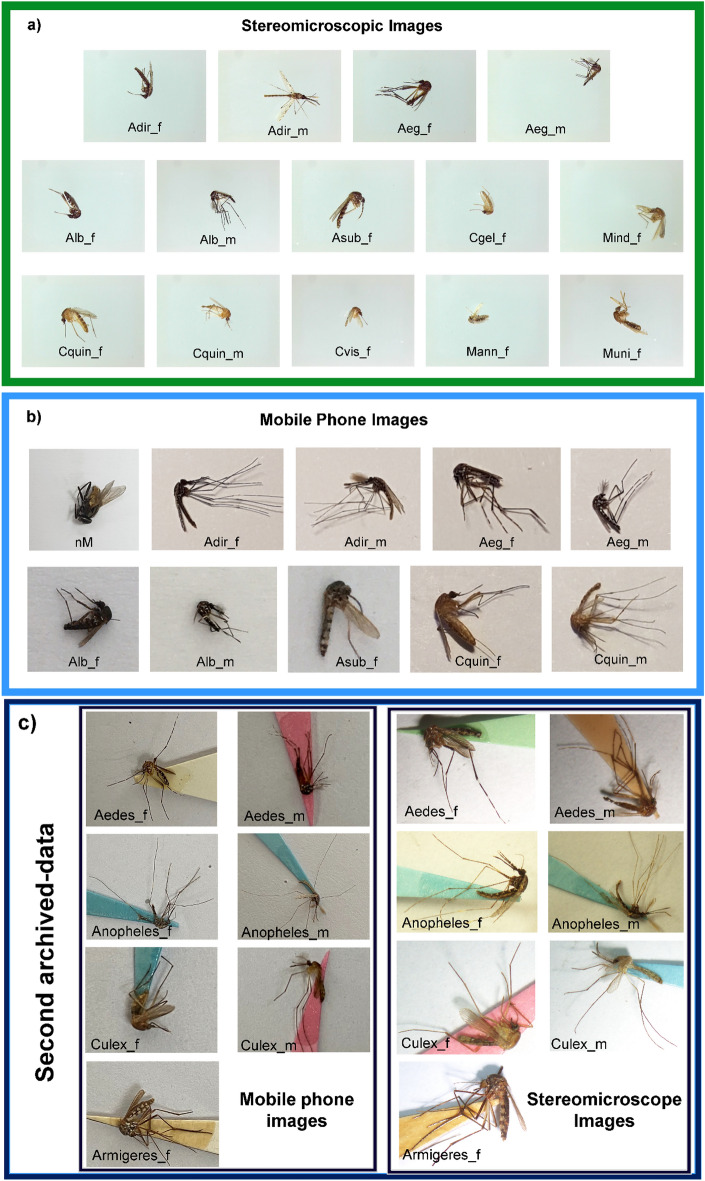
Dataset collection. Image-sets used were captured by a stereomicroscope and mobile phone cameras. Two-different sources were used including: (**a**) archived mosquito samples were captured by a stereomicroscope, (**b**) the same archived samples as described above were captured by mobile phone’s cameras. Those archived samples obtained were collected from four-different provinces in Thailand^16^. (**c**) archived mosquito samples obtained from Faculty of Veterinary Science, Chulalongkorn University^49,50^, were also captured by both stereomicroscope and mobile phone’s cameras.

A total of 7682 images, of which 4709 and 2973 images were collected from the microscope (Fig. 1a) and the mobile phone (Fig. 1b), respectively. Both two-datasets as described above were randomly assigned into training/ validation (90%) and training sets (10%) (Table 1). There were fifteen-classes of animal species including field-captured mosquitoes that had deformations in their body parts and had lost their characteristics leading to the variety of the datasets. A mainstream of image collection is mainly taken side-, upper-, and ventral views for training the neural network model^51^. The pixel densities of captured images is 2268 × 4032 and 2592 × 1944 pixels obtained from the mobile phone- and the stereomicroscopic images, respectively. On the basis of data from previous studies, it confirms the concept that the size of image resolution for machine learning is at least 320 × 320 pixels^52,53^. Although the use of different image resolutions was used to learn the neural network model, as seen above, their pixel densities were high enough for further training and evaluation of the proposed neural network models.

The assigned insect-specific characteristics were used to train/ validate a hybrid two-stage model based on a single deep-learning model of object detection and another, deep metric learning (DML), respectively. The dataset used is assigned for learning the You Only Look Once (YOLO) neural network in order to localize and also classify an animal species. These image sets of each class were labeled on the basis of a rectangular box (ground-truth labeling) and normally limited their potential environment as the region of interest (ROI). A threshold of probability was the confidential value obtained from this equation of Confidence = Pr(Object) + IOUTruthPred. Species-specific mosquitoes were corrected depending on the bounding box and were cropped to be a single mosquito per image by using our in-house CiRA CORE program. The ground truth conducted by entomologists under the CiRA CORE platform were publicly available from the GitHub repository with the url: https://git.cira-lab.com/cira/cira-core, based on the species of relative mosquito. The cropped images were then used as input for classifying their relative genus, species and gender by using deep metric learning networks.

### Experimental design for classification based DML

In this section, we have set three experiments including (1) miner comparison in order to find the best data mining procedure, (2) comparison trained-models of differential image sources, and (3) testing the trained model with unseen data collecting from another field study, which help confirm the model performance toward the robustness in real situation as follow;

(I) Data miner comparison:

We firstly study by using the most suitable model, Resnet-34^54^ as the neural network backbone. Comparison of the miners, which is important to define the positive- and negative-samples before embedding the feature vector onto 2-dimensional space. We applied all five-mines including AngularMiner, DistanceWeightedMiner, MultiSimilarityMiner, PairMarginMiner and TripletMarginMiner, respectively^36,37,55^.

(II) Learning conditions with different-image sources:

We then performed three-independent model training based on the optimized learning condition as described above. We set for three vectorized features extraction and independent learning conditions depending on types of image sources such as mobile phone datasets, stereomicroscope dataset and the combination of both sources (Supplementary Fig. S1). A quality performance for well-trained models would be evaluated by using the testing set which randomly split from both sources as described above.

(III) Robust trained-model with independently unseen dataset:

This section was designed for measuring the robustness of the best trained-model with optimized learning parameters. The quality performance of it was assigned whether the proposed neural network can be used to identify the independently unseen images collected from one another source of samples. The sample were previously prepared with sticky paper and set with a pin (Fig. 1c). Genus and gender levels of each animal sample were identified based on standard taxonomic key before capturing its image by experts who worked at faculty of Veterinary Science, Chulalongkorn University. The captured images with varied pixel resolutions were obtained from three-different mobile phone cameras. An individual sample was placed on a gray colored background and used 2× levels of zoom in. Of which, 716 images from four genus and were used. These images were rescaled to 32 × 32 pixels before using to be the query image in our CBIR process with 20-returned images from the database. All seven classes were divided into 10% for testing and the rest, 90% for pseudo-training data. The pseudo-training data were assigned and combined with previous trained data, but the new combination data won’t be trained with any pre-trained model, nevertheless, the CBIR-based prediction has done by previous optimized model.

### Development of deep neural networks

#### Object detection

The objective of this part was to find the suitable model for classification and localization of every single mosquito by using Yolo tiny-v4 neural network models from the in-house CiRA CORE platform (https://git.cira-lab.com/cira/cira-core). The one-stage model applied for helping us detection and collection based the export-crop module to be a single-mosquito per image. To prevent overfitting with feature variation of each class, data augmentation conditions were applied before model training as follows;

1. four-degree rotational angle increment as 45 steps at rotational angles (every 8 degrees) between minimum and maximum [− 180 to 180],
2. ten-percent improvement in brightness/contrast condition for every 0.2 stage (with a variance of ± 25 percent) between 0.4 and 1.2,
3. nine-steps of Gaussian blur conditions were adjusted for nine steps at each step, and
4. nine-steps Gaussian noise conditions were corrected for ten steps at each step.

For model training and evaluation, it was run on an Nvidia RTX2070 GPU platform. Learning rates were set at 0.001, which was assumed by the trained weight, reaching optimal accuracy versus loss. For the YOLO tiny-v4, the qualified models were trained for at least 100,000 epochs to record the learned parameters. The true positive value was considered by the likelihood of a threshold greater than and equal to 50%, nevertheless, the false positive values from the classification result are unexpected in medical diagnosis^56,57^.

#### Deep metric learning model

**Figure 2:**
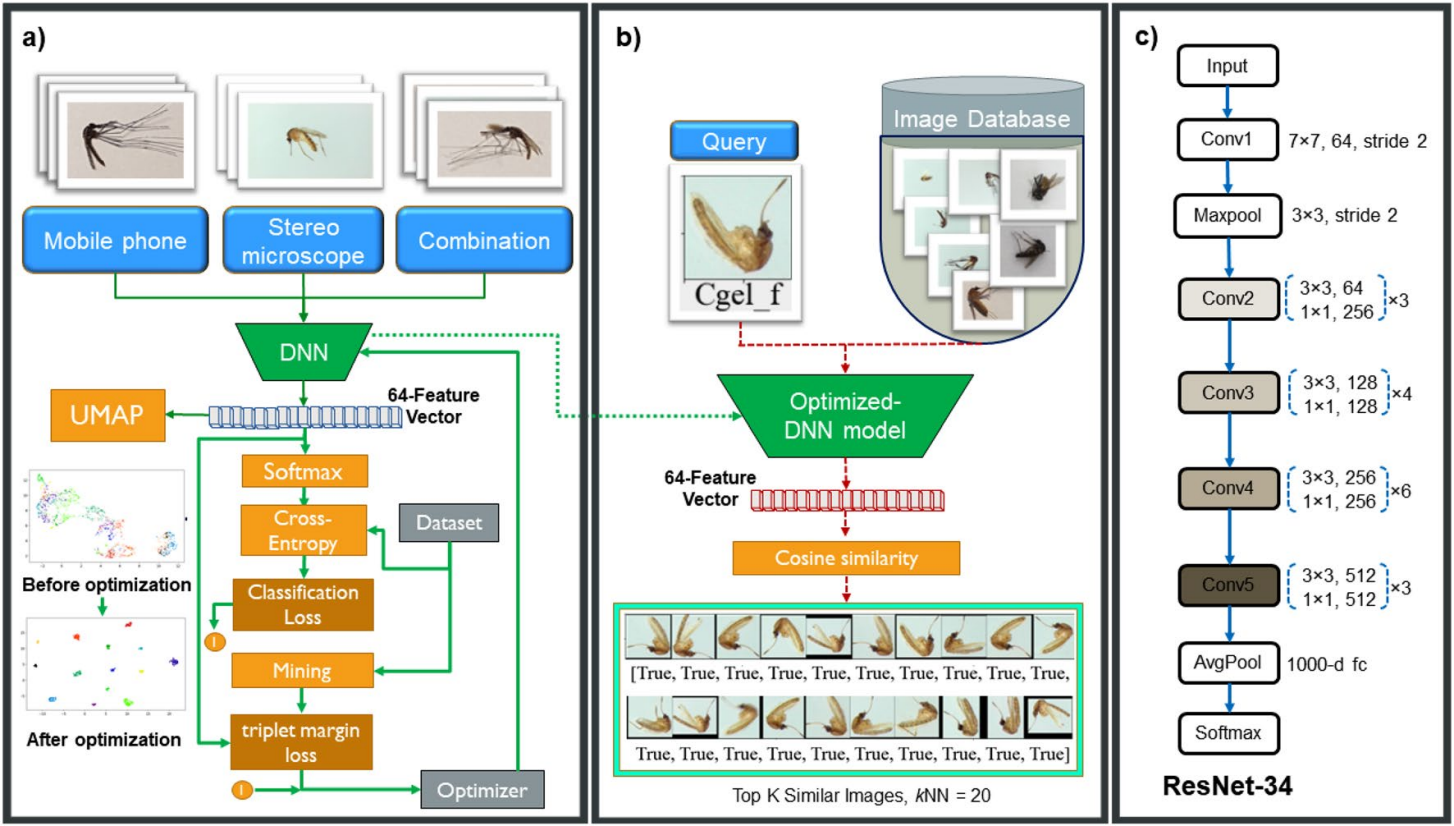
Architecture for learning approach. (**a**) Training phase were assigned by using three data, namely mobile phone, stereomicroscope and combination, respectively. (**b**) The testing phase with a query image based the content-based image retrieval was shown. (**c**) Resnet-34^54^, network architectures for Resnet-34 residual with 34 parameter layers (3.6 billion FLOPS), due to shortcuts increase dimensions within the architecture.

Before training, all three datasets were assigned including of the 1st, 2nd, and 3rd data are the mobile phone’s camera-captured images, the stereomicroscopes captured images and the combination data mentioned as above, respectively. The architecture of the training model of deep metric learning (DML) used is the Resnet-34 neural network under which default parameters were selected including the Cross-entropy loss function for classification, miner function, sampling strategy, and triplet-margin loss for embedding vector onto space, respectively (Fig. 2a,b). All processes including training, inference and evaluation of DML model were described in the pseudocode provided (Supplementary Fig. S2).

In this study, we applied the triplet margin Loss consisting of positive-, negative- and an anchor sample which was prepared from the miner function selected. The margin was calculated for identifying the positive or the opposite one as the negative. The positive sample locates within the border zone of the anchor, but the negative sample is vice versa. This distance value between anchor and positive (d_ap_) pair was small and less than a calculated margin. Nonetheless, the distance value between anchor and negative pair (d_an_) was greater than the margin. The formula of the triplet margin loss was shown as follows:

1 $$ L_{triplet} = \left[ {d_{ap} - d_{an} + m} \right]_{ + } $$

where the desired difference (d_ap_) and (d_an_), margin (m) = 0.1 was used as default in this study.

The DML model was designed into the three consecutive steps: including backbone, embedding and classifier parts. The pre-trained models on ImageNet, as backbone neural networks of the Resnet-34 models (Fig. 2c), were used as feature extractors in model training. Once the last feature layer was done, the important feature was transformed to the embedding space. During the experiment, the 1000-class output layer with a 64-dimensional embedding layer was set as the model embedder. Then, the embedding space classification was carried on by using *k*-mean clustering with *k* = 20, accompanying the ground truth label of the training dataset. According to the loss function within this step, the embedded layers kept the similar query input image to be closer and the dissimilar one to be far apart from each other^58^. At the end of the embedding layer, the last classifier layer was applied to support the trainer. Within an output, therefore, the dimensional vectors were given to the desired dimensions of the classifier as 20 groups.

The mining and sampling process, other two-main parts of the metric learning architecture, were considered to find the best samples while training. The Multi-Similarity Miner is used in this study, facilitating the production of the best pair mining candidates based on pair-based loss. Besides, it helps produce the optimal triplet mining by using the triplet loss during the model training. The loss will then be calculated based on those pairs or triplets. The Multi-Similarity Miner calculated the loss values of either pair or triplet values by setting the default epsilon of 0.1 to select the positive pairs or negative pairs^55^. This study, the M-Per-Class Sampler with the batch size of 16 and the number of samples per class is 8^59^. Training split was done at the 241 embedding batches due to the length of iteration is 7706 per epoch. Within the training process the sampling strategy is used to solve the random sampling problem, causing slow convergence and less performance of the model.

All five data miners studied come into two-steps including: (1) subset batch miners as for taking a batch of N embedding data and returned a subset n data to be used by a tuple miner or a loss function. (2) Tuple miners would take a batch of n embedding data and return k pairs/triplets to be used for calculating the loss function. Almost current miners are tuple miners that provides output as anchors, positives and negatives.

The combination models the DML mentioned above were trained on the Visual Studio Code version4, respectively. We trained the model based on Ubuntu version 16.04, 16 GB RAM, and NVIDIA GeForce RTX2070 graphic processor unit (GPU). All DML models obtained from open source PyTorch Metric Learning Library^59^. Training was performed on the visual studio code and the model deployment is under NVIDIA GeForce RTX2070 GPU. Each experiment consisted of 200 epochs. The best-trained models with their accuracy were collected automatically. Adam optimizer with the default parameters: β1 = 0.9, β2 = 0.999, weight decay = 0.001, epsilon = 10^−8^ and learning rate (0.00001 for backbone and 0.0001 for embedding and classifier) was applied. The output with 64-dimensional vectors embedded to be classified as a 20-dimensional feature vector.

### Evaluation of model performance

The trained models were evaluated for their quality performances by using an inference as described below. We presented these sections in two main parts: including inference and evaluation.

#### Inference

The inference of the trained-DML model was performed for known-image retrieval and clustering analysis against query images. Aligned with those, the well-trained model is also associated with the inference process since the evaluation of the error value obtained by optimizing the weight of the dataset during the training. Unlike the training process, the inference does not re-evaluate the output results. Likewise, the model training, the inference model employed the loaded-trained model and the match finder function, to do the matching pair on input embedding space by computing pairwise distances by using the Cosine Similarity function within its threshold of 0.5 in the testing phase. The *k*-nearest neighbor classifier (*k*NN) was finally facilitated to reconstruct the trained-dataset index and be beneficial for the similarity search based on the chosen distance metric. In this study, the inference is established on the Pytorch library^59^.

#### Model evaluation

The evaluation of the well-trained DML model is performed based on the nearest neighborhood image under the image-retrieval process against the query input. We set *k*NN = 20, the 20-nearest images against the query image returned. The quality performance of the proposed models was evaluated by several statistical parameters including: precision, sensitivity, accuracy and specificity^60^. The formulas for these parameters were shown as:

2 $$Precision = \frac{Tp}{Tp +Fp}$$

3 $$Sensitivity (Recall) = \frac{Tp}{Tp +Fn} $$

4 $$Accuracy = \frac{Tp +Tn}{Tp +Fp +Tn +Fn}$$

5 $$Specificity = \frac{Tn}{Fp +Tn}$$

6 $$F1 score=2\times \frac{recall \times precision}{recall+precision}$$

where Tp is the number of true positive classifications, Tn is the number of true negatives, Fp is the number of false positive classifications, Fn is the number of false negatives.

All statistics obtained from the confusion matrix are used to calculate the performances of the proposed model as described above. The predictions scores of each class are obtained from the number of corrected images retrieved from the nearest images from trained-database, converting to percent (%). The given class with the highest score would be considered as the predicted class of the query image. Then, the number of corrected images of the testing dataset were collected for constructing the confusion matrix table.

In addition, the performance of the proposed model was assessed by calculating the area under the receiver operating curve (ROC) with 95% confidence intervals (CIs) and the area under the curve (AUC) to determine the accuracy of the model’s using python. The ROC curve was plotted on the basis of the likelihood value of the 5% increment relative threshold. The 95 percent CIs is measured using a non-parametric bootstrap approach of 1000-fold image re-sampling.

## Results

In this study we have designed our experiments to find the optimal training conditions for model learning including (1) using different sources and location of datasets in order to study the model as robustness, (2) integrating object detection and DML and the CBIR process, and (3) optimizing the training condition for DML. Within the DML we find our best data Miner from the comparison designed. The hybrid two-stage neural network model was developed based on independent-two algorithms, namely, object detection and another, deep metric learning. The best-selected Yolo tiny-v4 and Resnet-34 models were optimized under the in-house CiRA CORE platform and another under Pytorch program, respectively. In this study, to solve the conventional classification problem, the deep metric learning model was employed and trained with a number of dangerous-mosquito species as follows:

### Data miner comparison

The data miner functions as an empirical section in the DML architecture by mining the positive- and negative pair sample and also calculates adjusted distance of those between those to anchor during the optimization process. Hence, the learning process performed by using a suitable miner could result in the best-selected trained models for further implementation.

**Figure 3:**
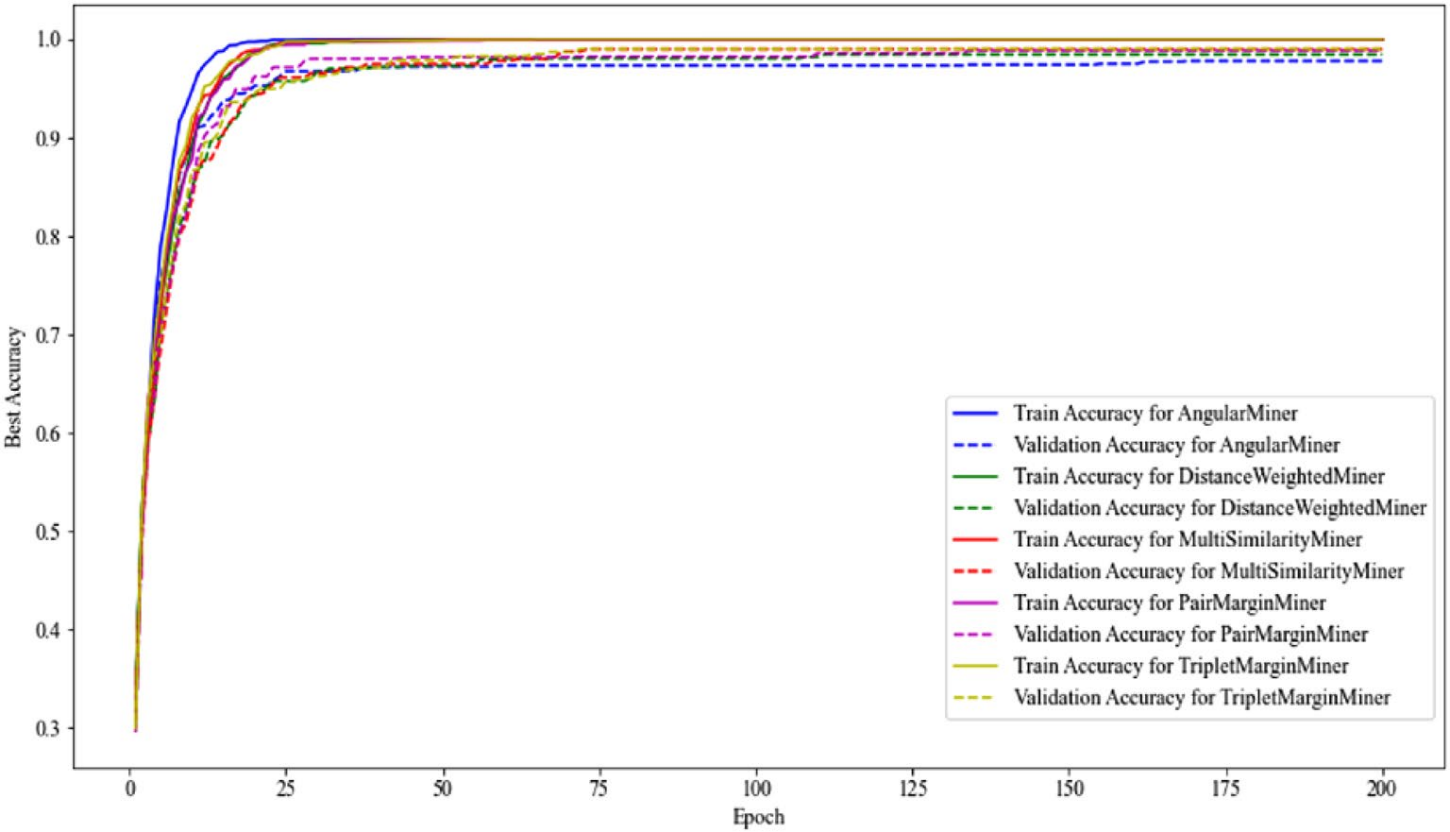
Training accuracy for five Miners comparison. The plots of training accuracy and validation accuracy of each data Miner are represented by a smoothed line and a dash line, respectively.

**Figure 4:**
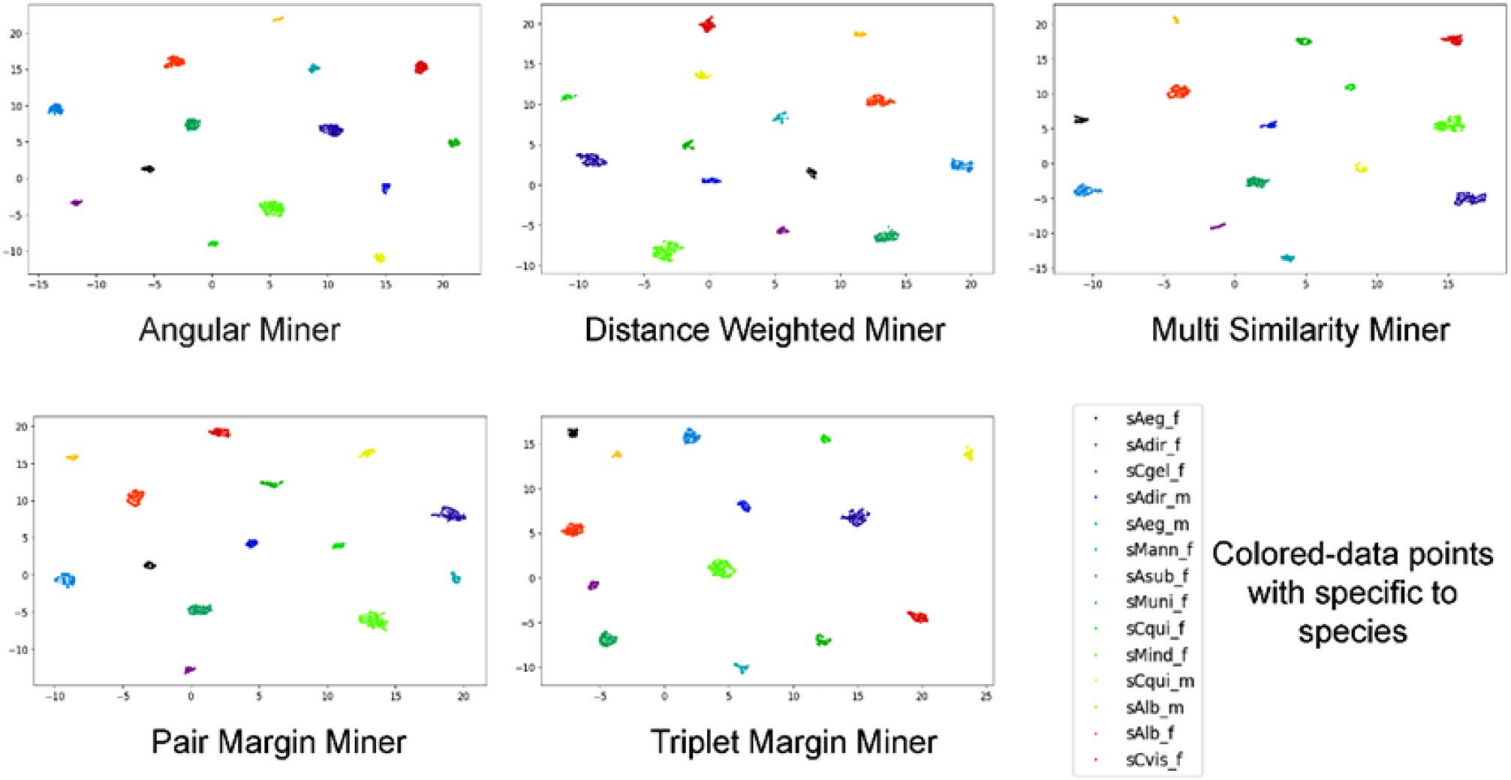
UMAPs for five Miners comparison. All five UMAP represented well-clustering representation of the dimensional reduction based five-data miner based trained-DML models. All data miners used comprise of Angular Miner, Distance Weighted Miner, Multi Similarity Miner, Pair Margin Miner and Triplet Margin Miner, respectively. Each class of mosquito species was an assigned by a single colored datapoint.

In this section we did a comparison of all five miners to find the most effective one including Angular Miner, Distance Weighted Miner, Multi Similarity Miner, Pair Margin Miner and Triplet Margin Miner, respectively (Table 2). Overall, all trained models with a single five-miner used showed similarly high-performance ranking of 98% to 100% for precision and sensitivity, and 99% to 100% of specificity and accuracy, respectively. Besides, optimized training models can be shown based on the plateau region of the training accuracy and validation curves which infer the model learning achievable with training data well, suggesting to avoid overfitting condition and is able to make accurate prediction based unseen data testing (Fig. 3). The result of dimensional reduction as UMAP representation with clear clustering data points within a relative class (Fig. 4). These helps confirm well-trained models for further predicting the testing data.

**Table 2:** Performance analysis of miner comparison based on 14 classes using image from stereomicroscope.

Types of miners	Precision (%)	Sensitivity (%)	Specificity (%)	Accuracy (%)
Angular miner	100.00	100.00	100.00	100.00
Distance weighted miner	100.00	100.00	100.00	100.00
Multi similarity miner	99.66	99.66	99.97	99.95
Pair margin miner	99.66	99.66	99.97	99.95
Triplet margin miner	98.99	98.99	99.92	99.86

The miners used to include Angular Miner, Distance Weighted Miner, Multi Similarity Miner, Pair Margin Miner and Triplet Margin Miner, respectively.

Considering species-specific evaluation, miss identification is found for both genders of *Aedes aegypti*, *Aedes albopictus*, *Culex gelidus* and *Culex vishnui*, respectively (Table 3). This may be due to testing the trained model with damaged and broken field caught samples leading to similar feature appearance between genders of *Aedes genus* and between species of the *Culex genus*. Nevertheless, the small proportion of an error classification found is under employed to the Triplet Margin Miner with at least 83.33% of precision for identifying male of *Aedes albopictus* (Table 4). Although the species is important for growing the mosquito population density, it is not crucial to transmit any mosquito-borne pathogens due to it having no blood-feeding in male. Therefore, the rest of the four-miners suggested the most suitable selectable-miners.

**Table 3:** Performance analysis of miner comparison based on 14 classes.

No	Species	Sensitivity (%)					Precision (%)				
		AM	DWM	MSM	PMM	TMM	AM	DWM	MSM	PMM	TMM
1	sAdir_f	100.00	100.00	100.00	100.00	100.00	100.00	100.00	100.00	100.00	100.00
2	sAdir_m	100.00	100.00	100.00	100.00	100.00	100.00	100.00	100.00	100.00	100.00
3	sAeg_f	100.00	100.00	100.00	92.86	100.00	100.00	100.00	100.00	100.00	93.33
4	sAeg_m	100.00	100.00	100.00	100.00	96.15	100.00	100.00	100.00	96.30	100.00
5	sAlb_f	100.00	100.00	100.00	100.00	93.55	100.00	100.00	100.00	100.00	100.00
6	sAlb_m	100.00	100.00	100.00	100.00	100.00	100.00	100.00	100.00	100.00	83.33
7	sAsub_f	100.00	100.00	100.00	100.00	100.00	100.00	100.00	100.00	100.00	100.00
8	sCqui_f	100.00	100.00	100.00	100.00	100.00	100.00	100.00	100.00	100.00	100.00
9	sCqui_m	100.00	100.00	100.00	100.00	100.00	100.00	100.00	100.00	100.00	100.00
10	sCgel_f	100.00	100.00	97.06	100.00	100.00	100.00	100.00	100.00	100.00	100.00
11	sCvis_f	100.00	100.00	100.00	100.00	100.00	100.00	100.00	95.83	100.00	100.00
12	sMann_f	100.00	100.00	100.00	100.00	100.00	100.00	100.00	100.00	100.00	100.00
13	sMind_f	100.00	100.00	100.00	100.00	100.00	100.00	100.00	100.00	100.00	100.00
14	sMuni_f	100.00	100.00	100.00	100.00	100.00	100.00	100.00	100.00	100.00	100.00
Average		100.00	100.00	99.66	99.66	98.99	100.00	100.00	99.66	99.66	98.99

Comparison of data miners varied including AM abbreviates for Angular Miner; DWM for Distance Weighted Miner; MSM for Multi Similarity Miner; PMM for Pair Margin Miner and TMM for Triplet Margin Miner, respectively.

**Table 4:** Confusion matrix for testing dataset.

Actual class														
Classes	sAdir_f	sAdir_m	sAeg_f	sAeg_m	sAlb_f	sAlb_m	sAsub_f	sCqui_f	sCqui_m	sCgel_f	sCvis_f	sMann_f	sMind_f	sMuni_f
Predictive class														
sAdir_f	**12**	0	0	0	0	0	0	0	0	0	0	0	0	0
sAdir_m	0	**15**	0	0	0	0	0	0	0	0	0	0	0	0
sAeg_f	0	0	**14**	0	0	0	0	0	0	0	0	0	0	0
sAeg_m	0	0	0	**26**	0	0	0	0	0	0	0	0	0	0
sAlb_f	0	0	0	0	**31**	0	0	0	0	0	0	0	0	0
sAlb_m	0	0	0	0	0	**10**	0	0	0	0	0	0	0	0
sAsub_f	0	0	0	0	0	0	**28**	0	0	0	0	0	0	0
sCqui_f	0	0	0	0	0	0	0	**12**	0	0	0	0	0	0
sCqui_m	0	0	0	0	0	0	0	0	**15**	0	0	0	0	0
sCgel_f	0	0	0	0	0	0	0	0	0	**34**	0	0	0	0
sCvis_f	0	0	0	0	0	0	0	0	0	0	**23**	0	0	0
sMann_f	0	0	0	0	0	0	0	0	0	0	0	**15**	0	0
sMind_f	0	0	0	0	0	0	0	0	0	0	0	0	**44**	0
sMuni_f	0	0	0	0	0	0	0	0	0	0	0	0	0	**17**

The kNN used is 20 returned images from database. The trained model-based Angular Miner. Significant values are in bold.

In this study, deep metric learning with a simple ResNet architecture (Fig. 2) can potentially outperform the classical cross-entropy classification problem using the same ResNet network due to several reasons:

1. Focus on Relative Distance: Metric learning focuses on learning the relative distances between different classes, rather than directly classifying them. This way, the model learns to discriminate better between classes, which can lead to better performance, particularly in tasks where inter-class variance is significant.
2. Better Generalization: Metric learning optimizes the model to ensure that the learned embeddings of the samples from the same class are closer to each other and far apart from samples of different classes. This approach can result in better generalization to unseen data, as the model is not focusing on the absolute features of each class but the relative features across different classes.
3. Beneficial for Large and Imbalanced Datasets: Deep metric learning models, such as those utilizing triplet loss or contrastive loss, often perform better with large and imbalanced datasets, which can be challenging for traditional cross-entropy classification models and
4. Handling New Classes: Metric learning models can handle new classes better than traditional classification models. With a metric learning model, if a new class is added, it doesn't necessarily require retraining of the entire model as the model is based on distance measures. On the contrary, a traditional classification model would require retraining from scratch or significant fine-tuning if a new class is introduced.

### Learning conditions with different-image sources

**Figure 5:**
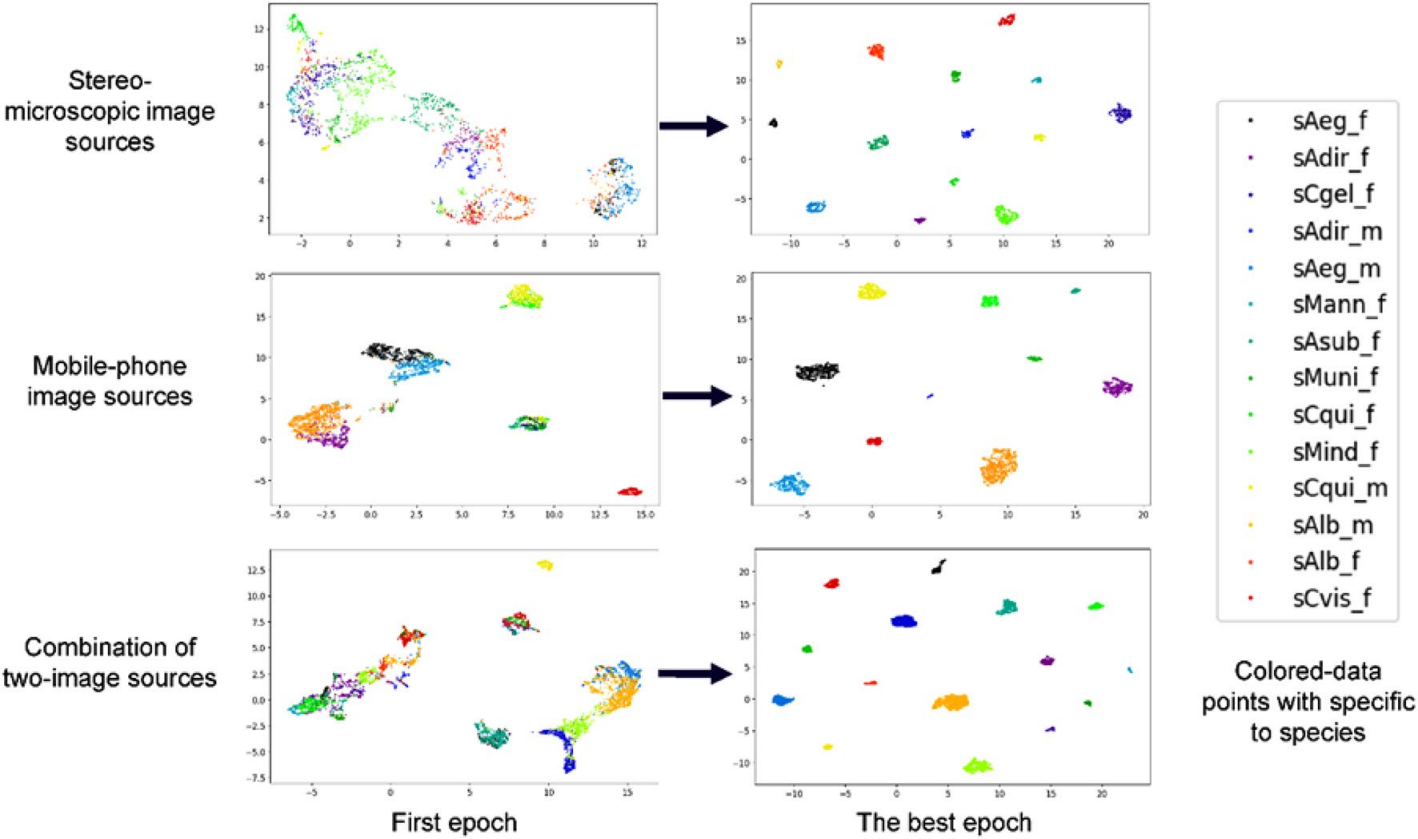
UMAPs for different image sources. The first and the best UMAP were compared. Above, middle and below ones are the plot of trained-models with stereomicroscope-, mobile phone-, and the combinations, respectively. Each class of mosquito species was an assigned by a single colored datapoint.

Since many suitable miners gave good enough results, we selected the Multi-Similarity Miner as a default parameter for developing the model with varied image sources including the stereo-captured images for 14-independent classes, the mobile phone captured images for 10-independent classes and the combination of both image sources for 15-independent classes as above (Fig. 1a, b). All three plots of training loss per iteration shown the optimized model using ResNet-34 backbone (Supplementary Fig. S3). Only the plot of the stereomicroscope dataset learnt model showed rarely fluctuated with large number of iterations, but the best trained weight file was automatically saved. Also, all dataset were combined for model training which results in more compact clustering analysis (Fig. 5) which inferring saturated and optimal condition observed. This is the advantage of the combination data, excepting for the separable first two data mentioned as above.

The trained-models with all three-image sources provided a high degree of greater than 99% for precision, sensitivity, specificity and accuracy, respectively (Fig. 5; Table 5). The UMAP results obtained from model learning with three different datasets showed clear clustering analysis based the best epoch when comparing to the first training epoch. In addition, data source wise comparison still showed well clustering among all classes. Interestingly, the combination sources-trained model rarely compact clustering than any others specifically in the orange cluster representing for male *Ae. albopictus*, the light-blue cluster for male *Ae. aegypti* and the blue cluster for male *An. dirus*, respectively. The rationale of supporting the presented result may be due to associate with a large amount of sample size used, giving more compact clusters belonging to the criteria to improve the model learning of supervised learning models (Fig. 5). Animal species-wise comparison of the trained models showed greatest performance when training the model with the stereomicroscopic image upto 99.66% for sensitivity and precision, respectively. Nevertheless, the trained model with the mobile phone images (*Anopheles dirus*, female and *Aedes aegypti*, female) gave lower than 90% of both sensitivity and precision (Tables 6, 7) that may be due to different sample size and also their quality of captured images used affected learning accuracy of the model used. Interestingly, the trained model with the combination of two-image sources showed an empirical performance with greater than 90% and 95% for sensitivity and precision, respectively (Table 6). Previous publication indicated that combination of multiple-data sources plays a role as exploring the possibilities of using the model to improve future data collection quality. Also, scalable multiple data for model learning significantly highlights the cost-effective monitoring of disease vectors, especially in the context of the recent emergence and re-emergence of mosquito-borne diseases worldwide^61,62^. As a result, combination also increase the clustering analysis in UMAP clear and compact as shown in Fig. 5. Our contribution is to develop and implement our deep metric learning approaches to classify on mosquito populations in multiple regions in Thailand by using the combined data, which is comparable to an augmented information. Hopefully, a framework provides the approaches to predict region specific mosquito species, which may be applied to other regions in tropical area near Thailand.

**Table 5:** Performance analysis of trained Resnet-34 model to test the differential images collecting from stereomicroscope, mobile phone and the combination of both sources.

Sources of dataset	Precision (%)	Sensitivity (%)	Specificity (%)	Accuracy (%)
Stereomicroscope	99.66	99.66	99.97	99.95
Mobile phone	99.15	99.15	99.91	99.83
Combination	99.22	99.22	99.94	99.90

**Table 6:** Performance analysis of trained Resnet-34 model for testing images collecting from the stereomicroscope, the mobile phone and the combination of both image sources.

No	Species	Index	Sensitivity (%)			Precision		
			Stereo	Mobile	Combine	Stereo	Mobile	Combine
1	Non-mosquito (*Musca domestica*)	mNM	–	100.00	100.00	–	100.00	100.00
2	*Anopheles dirus*, female	Adir_f	100.00	88.89	90.48	100.00	100.00	95.00
3	*Anopheles dirus*, male	Adir_m	100.00	100.00	99.01	100.00	100.00	100.00
4	*Aedes aegypti*, female	Aeg_f	100.00	94.44	96.88	100.00	89.47	96.88
5	*Aedes aegypti*, male	Aeg_m	100.00	100.00	100.00	100.00	100.00	97.62
6	*Aedes albopictus*, female	Alb_f	100.00	100.00	98.61	100.00	100.00	100.00
7	*Aedes albopictus*, male	Alb_m	100.00	98.21	100.00	100.00	98.21	97.06
8	*Armigeres subalbatus*, female	Asub_f	100.00	100.00	100.00	100.00	100.00	98.90
9	*Culex quinquefasciatus*, female	Cqui_f	100.00	100.00	100.00	100.00	96.97	100.00
10	*Culex quinquefasciatus*, male	Cqui_m	100.00	98.21	100.00	100.00	100.00	100.00
11	*Culex gelidus*, female	Cgel_f	97.06	–	100.00	100.00	–	100.00
12	*Culex vishnui*, female	Cvis_f	100.00	–	100.00	95.83	–	100.00
13	*Mansonia annularis*, female	Mann_f	100.00	–	100.00	100.00	–	100.00
14	*Mansonia Indiana*, female	Mind_f	100.00	–	100.00	100.00	–	100.00
15	*Mansonia uniformis*, female	Muni_f	100.00	–	100.00	100.00	–	100.00
Average			99.66	99.15	99.22	99.66	99.15	99.22

**Table 7:** Performance analysis of trained Resnet-34 model for testing images collecting from the combination of the second sources, stereomicroscope and mobile phone cameras.

No	Genus and gender’s levels	Combination				Stereomicroscope				Mobile phone			
		Sensitivity (%)	Specificity (%)	Precision (%)	Accuracy (%)	Sensitivity (%)	Specificity (%)	Precision (%)	Accuracy (%)	Sensitivity (%)	Specificity (%)	Precision (%)	Accuracy (%)
1	Non-mosquito (*Musca domestica*)	100.00	99.87	96.67	99.88	100.00	100.00	100.00	100.00	100.00	99.81	96.67	99.82
2	*Anopheles*, female	89.66	99.62	89.66	99.27	100.00	99.14	84.21	99.71	76.92	99.82	90.91	99.30
3	*Anopheles*, male	99.04	99.86	99.04	99.76	100.00	99.71	94.74	99.72	98.84	99.79	98.84	99.65
4	*Aedes*, female	96.28	98.89	96.28	98.29	94.44	97.41	86.44	96.97	97.01	99.77	99.24	99.12
5	*Aedes*, male	99.09	99.44	96.46	99.39	94.87	99.38	94.87	98.90	100.00	99.60	97.26	99.65
6	*Armigeres*, female	97.96	99.59	96.97	99.39	90.63	99.70	96.67	98.90	100.00	99.40	95.65	99.47
7	*Culex*, female	88.89	100.00	100	98.42	89.74	99.30	97.22	97.25	84.62	100.00	100.00	98.95
8	*Culex*, male	98.57	99.07	90.79	99.03	80.95	99.71	94.44	98.62	98.18	98.83	90.00	98.77
9	*Mansonia*, female	100.00	99.73	97.44	99.76	100.00	99.30	97.44	99.45	100.00	100.00	100.00	100.00
	*Average*	96.61	99.56	95.92	99.24	94.52	99.29	94.00	98.78	95.06	99.67	96.51	99.41

Data combination from different sources performed in order to increase variability of data and see this variation of them would have no affection to feature learning during cross-testing of the proposed model. In addition, the combination of different sources of data could technically improve the classification and refinement of the deep learning method^61^. Hence, increasing data volume is unnecessary.

**Figure 6:**
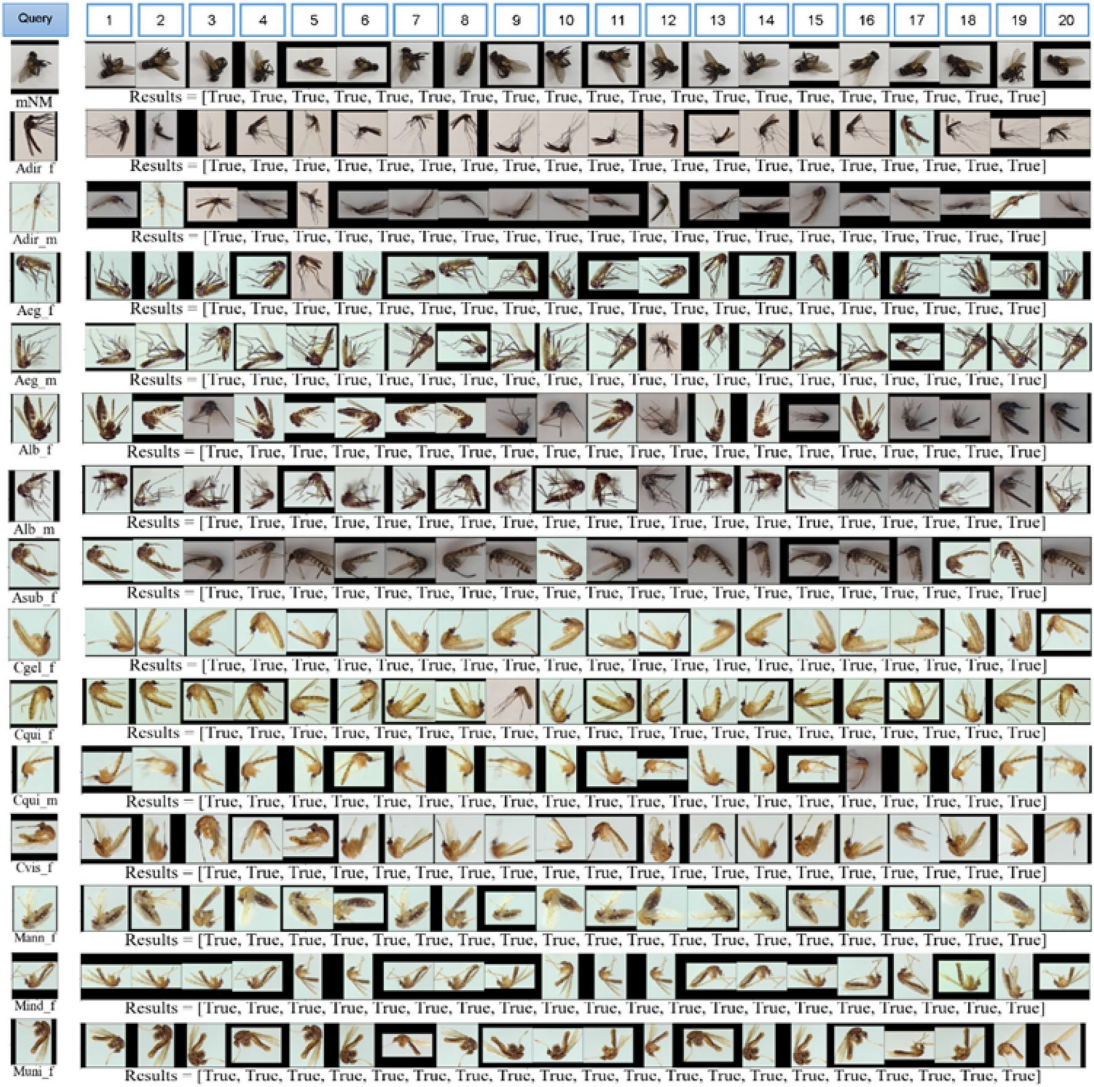
CBIR for single- and combination testing data.

Although the damaged samples with loosen scales and discoloration which was specifically undistinguishable by naked-eye, were used, the trained model can also discriminate with small amounts of misidentification. There are only two false negatives in *A. dirus*’s female and *Ae. albopictus*’s female and one false negative in *A. dirus*’s male and *Ae. aegypti*’s female, respectively (Suppl. Tables). At the 20 retrieved images are given comparing to their feature to the query image, unseen testing data. The similarity between the unseen testing image and the database was measured by Euclidean distance. The first left-side retrieved image is the most similar but, the second is less similar and so on (Fig. 6). As a result, even though different learning with varied image sources, deep metric learning gave superior performance representing that classification problem can be solved by the DML model as effectively (Fig. 6). In addition, all high auROC values also supported the evaluation metrics found in Table 5 and those greater than and equal to 0.996 for all trained models (Supplementary Fig. S4).

We compare the performance results between the DML model with voting system (CBIR, kNN = 20 returned images) and the model with no voting system (kNN = 1 returned images). Several evaluation metrics were used to assessed the trained models including accuracy, specificity, precision, recall and F1 score, respectively (Supplementary Tables S7–S9).

The model performance trained with the mobile phone dataset shows comparable results between k = 1 and k = 20 (Supplementary Table S7). On average, although there is contrast result between precision and recall, the harmonized mean (F1 score) between those metrics gives very similar values of 0.983 (k = 1) and 0.982 (k = 20). Surprisingly, the performance of the model with voting system using the stereomicroscope dataset provided higher metric values than that of non-voting system (Supplementary Table S8). The similar trend of the combination to the mobile phone dataset were analyzed (Supplementary Table S9).

Although the numbers of class labels were studied, of which, the classification power of using the proposed voting system can also be applied to obtained the correct answer. Comparing to the classification algorithms that need a large amount and class-balanced data with a unique feature for training the classification model, their results depending on the % probability. As a result, the CBIR system seems to be appropriated for classifying unseen data with the small sample size, unbalancing class even the closer intra- and inter-class variations^63,64^, for instance, the stereomicroscope data as described in Table 1.

### Robustness of the trained-model with independently unseen dataset

Our best selected neural network model was then used to validate its performance with unseen dataset obtained four-animal genus and assigned for seven classes (Table 7). All image sets used were collected by using the independent mobile phone camera and also the stereomicroscopic cameras, which are given the varied pixel-resolutions of the images. In this section, the proposed model was challenged with extremely uncontrolled environmental factors such as degree of lighting, image scales, background colors and zoom levels even though those factors described above were assigned to be controlled (Fig. 1c).

**Figure 7:**
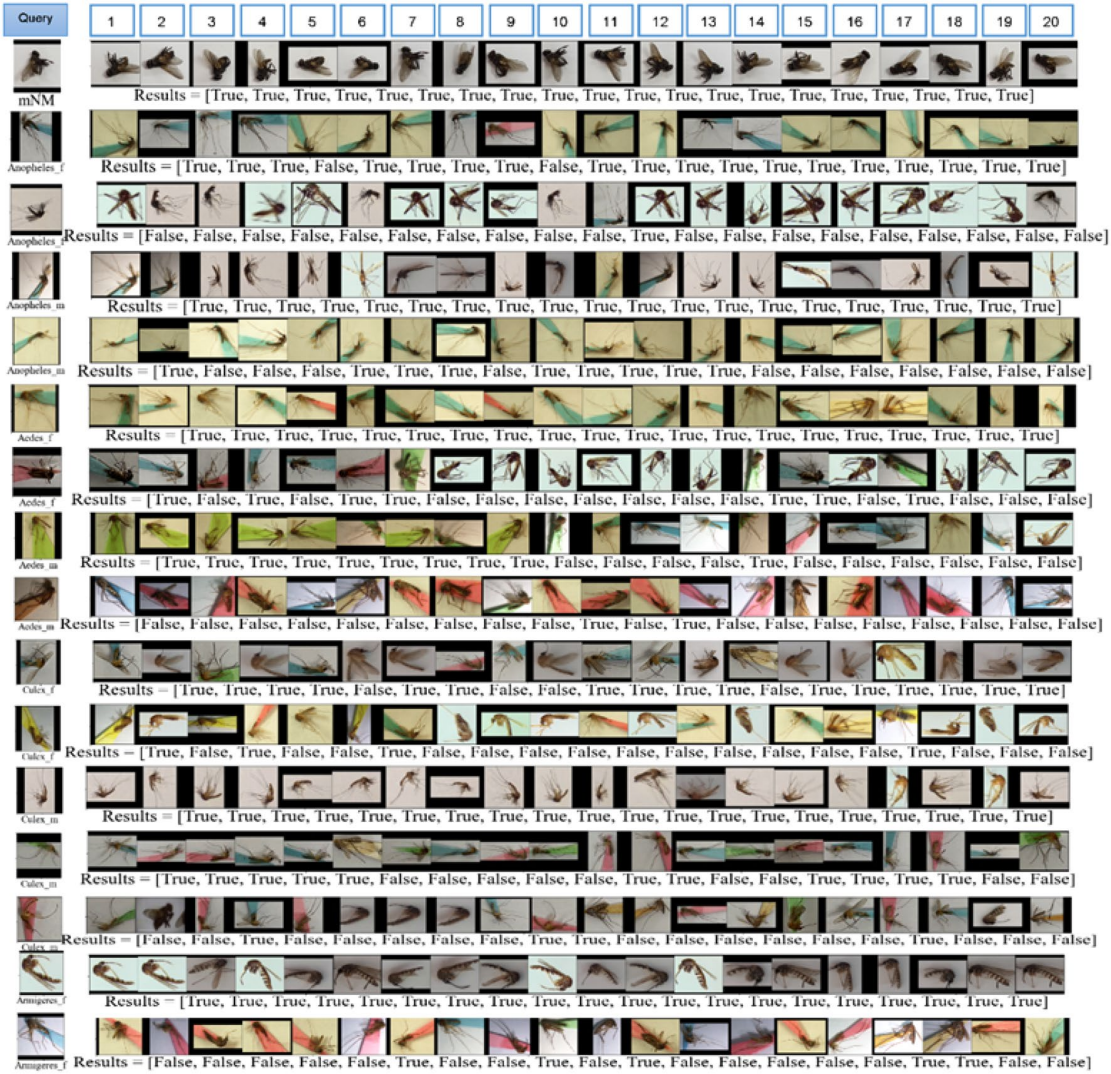
CBIR for second testing dataset.

We recruited more data from different sources to determine whether the trained model can be a good enough to classify complexity and a flood of information in open-world image data (Fig. 7). As a result, overall qualitative performance of the best model, based on the CBIR with kNN = 20 (Fig. 7), revealed an outstanding model with specificity for 99%, accuracy for 99%, sensitivity for 96% and precision for 95%, respectively (Table 7). Also, camera-wise comparison showed similar results. Here was the robustness model which presented in the CBIR result and the model used with no re-train with a new sample collected, assigned pseudo labels.

**Figure 8:**
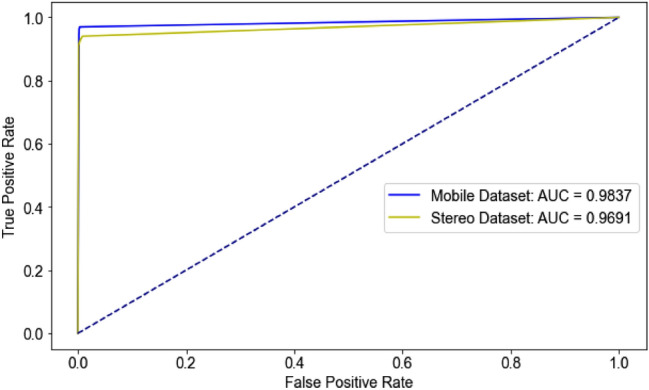
ROC curve for second testing dataset. Two different cameras used to collect the same samples. The cameras are stereomicroscope and one another, mobile phone cameras, respectively.

Although superior average performance of the proposed model for identifying the genus and gender was measured, true positive prediction data found less than that in previous the first data source, but the false negative data were increased, specifically for *Anopheles* (female), *Aedes* (female) and *Culex* (female) due to their potential area contained the color-pinned papers during image capturing (Suppl. Tables S4–S6). Nevertheless, the research result seems to be possible prediction due to uncontrolled environmental factors suggested those as before. In addition, although the low prediction result obtained when comparing to previous result with first data source, the model still reveals the outstanding with auROC greater than 0.960 for both image data (Fig. 8), which supporting the learning system both practical and empirical model. Therefore, the trained model could help solve the classification problem of the entropy in real-world data.

Overall, the proposed model can be used to identify many mosquito vector species, such as *Aedes*-, *Anopheles*- and *Culex* mosquito vectors, which could contribute to the control measure and employ toward the vector management in the realistic situation.

## Discussion

In the research study result of the DML-based CBIR process showed great success for a new identification challenge for mosquitoes of public health concerns that can transmit various mosquito-borne pathogens, including dengue virus, ZIKA virus, West Nile virus, filaria and also malaria parasite in both animals and humans. Previously, a Machine Learning (ETC model) and Deep Learning (VGG16) were used to classify two critical disease-spreading classes of mosquitoes, *Aedes *and *Culex*. Limitation focuses on two critical disease-spreading classes of mosquitoes, *Aedes* and *Culex*, and does not consider other species^17^. However, our study has been investigated with greater number of mosquito species where distributed in Thailand. Hopefully, the proposed model would be challenged in several fields to gain more data training. Variation and special characteristics of the animal species used enables the CBIR system to operate with outstanding performance metric up to 99% for developed model and also greater than 95% in identifying the unseen second source of the image data. Our result revealed higher accuracy relative to other mosquitoes^9,11,65,66^. As a previous study, using a large and annotated-data could improve model efficiency for uncommon image classes^9,67^. Mwanga et al.^30^ showed high accuracy to ~ 98% accuracy for predicting mosquito age classes based on the dimensionality reduction and transfer learning techniques, that help confirm that the advantage of the similar techniques used as obtained in our study.

In this study, the optimized deep metric learning approach demonstrated its performance in helping solve the classical classification problem by making-decision for answer based on the returned image from trained dataset. Good success in distinguishing between dangerous mosquitoes and non-mosquito (total 15 classes) achieved high accuracy approximately 99% in the Miner-wise relation. The results need to be validated with unseen testing data with varied environmental factors. Although the performance of the proposed model testing with image data obtained from the second source gave lesser than 90% in sensitivity and precision for malaria vector (*Anopheles*) and West-Nile virus vector (*Culex*), average performance of our trained model still showed excellent (Suppl. Tables S1–S6). Additionally, we applied three different levels of Gaussian noises to three female mosquito species, namely *An. dirus*, *Ae albopictus* and *Ar. subalbatus*, respectively. As a result, the AUC under the ROC curve gradually reduced along with increasing noise degrees as expected (Supplementary Fig S5). Also, varied AUCs between animal species found may be depended on variation of biological data studied.

In this study, we normally have *Anopheles dirus* as one of main vectors for malaria in humans and animals, *Aedes Aegypti* and *Ae. albopictus* as main vector for dengue and *Culex quinquefasciatus* as secondary vector for dengue^16^, and *Cu. vishui*, *Cu. gelidus* and *Cu. tritaeniorhynchus* as a vector for Japanese Encephalitis^68^. There are several possible secondary vectors for malaria (*An. nivipes*, *An. philippinensis, An. barbirostris, An. lesteri and An. annularis*), dengue (*Ae. scutellaris*) and Japanese Encephalitis (*Ae*. *j*. *japonicus*)^69^. Although there are only 14 classes presented in this study, the proposed model can be shown the generalized approach to deal with several species of the mosquito vector in Thailand. Further study, development of deep metric learning approach with possible secondary vector could increase the potential AI platform to challenge wide range of populated mosquito vector in Thailand.

As the result obtained, this model can be useful in automatic surveillance of dangerous mosquitoes in remote areas. The predictions can also be extended to entomologically related work, as all organisms could be identified with high confidence using the proposed network model. This is because the dataset used quite covers a wide range of mosquito species that live in tropical countries where the mosquitoes are often responsible for the spread of several diseases in humans and animals^16,70–73^. Similarly, the publication introduces the use of Deep Metric Learning models, for the classification of mass spectra of 12 malaria mosquito species and 18 tick species. Different backbone use comparing to our study (using ResNet), the study demonstrates the effectiveness of Siamese Neural Networks (SNNs) and Triplet Neural Networks (TNNs) in accurately and efficiently categorizing mass spectra. The model performance of using the proposed three algorithms mentioned as above ranged from 94 to 99% for mosquitoes and from 91 to 93% for ticks, respectively. This also help confirm the achievement to classify the medical insect species by using DML techniques. However, the study does not compare the performance of the Deep Metric Learning models to other classification methods, which could provide further insights into the effectiveness of these models^31^.

Deep metric learning is the combination between deep learning and metric learning, in which the model used aims to extract and learn object features as multidimensional features vectors (representing the distance and locations). The two similar features vectors were assigned in a neighboring space that have similar features. This is because the distances between them are minimized^48^. Considering the different aspects between deep metric learning and deep learning techniques, deep learning attempts to define characteristics of each object’s class based on percentage of probability, nevertheless, deep metric learning learns to measure the similarities between object within any class by generating an embedding datapoints in latent space where similar features of any object locate closer in low-dimensional space. Here may be the concept learning to support excellent result obtained in our study. Interestingly, the distinguishable power of well-trained DML module can be used to describes embedding features with both the closer intra-class and discriminative inter-class variations. This is because the features were better generalized enough, though the unseen classes recruited.

Although computational modeling has had a significant influence on science work, more enhancements are needed. For example, it requires (1) a large number of training details and intact samples, and (2) a new methodological architecture to be learned and managed data collected from different cameras^74^ and also the difference in focus quality may make it difficult to label datasets and train models^75^. Using the same basic type of camera property and/ or stereo microscope to capture the mosquito image could help promote further deployment of the embedded device network concept in remote areas elsewhere, without re-training the data prior to use in real-time scenarios. Deep metric learning approach is suitable to deploy into current surveillance and control measure of the entomological work.

## Conclusion

We obtained archived samples from two different study sites and represented to national-level data. The first study site, the sample was collected from four provinces in Thailand including Kalasin (Northeastern region), Bangkok (Central region), Prajaubkirikhan (Southern region) and Chonburi (Eastern region), respectively^16^. The second study site, archived samples were obtained from Kanchanaburi province, the western region of Thailand. The proposed DML network algorithm and the CBIR provides great potential for newly automatic screening and/ or support embedding devices for entomological staff during mosquito identification. We have achieved the DML model developments using the ResNet-34 and the embedding feature vector relied on the triplet-margin loss as feature-vector embedding function. The model can be learnt two new generated data, captured by stereomicroscope, mobile phone’s cameras and also combination of both two data mentioned as above. The 20-top rank of retrieval images-based the k-nearest neighbors showed the suitable process for testing entomological image gave high values of both the true positive and true negative rate^76^. Variation task of biological samples has been solved and accomplished by encouraging them to analyze the image sample based Euclidian distance similarity between the query and dataset as being the same as the model test set. Due to DML is type of supervised learning model that the great performance of it depending on a large sample size and variation of the image dataset. The preparation of image dataset would be achieved if there are (1) the intact mosquito samples were used. The color and pattern of mosquito anatomy are found such as proboscis and palpi, terga and abdomen, mesonotum, femur and tarsi, respectively^5^. Next, (2) angles taken of mosquito images including lateral-, dorsal- and ventral sides, the more position collected, the greater performance of training model obtained. (3) Image size collected by different quality of cameras can affect the trained model during testing in real world^77^. In this context, the CBIR-based trained DML algorithm achieved state-of the-art performance on real world data^78^, giving robustness model on independently unseen dataset collected from other study site.

## Supplementary Information

Supplementary Information.

## Data Availability

The data that support the findings of this study are available upon request to the corresponding author.
