# Supplementary material for: Automatic identification of medically important mosquitoes using embedded learning approach-based image-retrieval system
Source: Sci Rep. 2023 Jun 30;13:10609. doi: 10.1038/s41598-023-37574-3 (PMC10313673; doi:10.1038/s41598-023-37574-3)
Supplement: Supplementary file 1 — Supplementary Information. [file 41598_2023_37574_MOESM1_ESM.docx]

**Automatic identification of medically important mosquitoes using embedded learning approach-based image-retrieval system**

Veerayuth Kittichai^1^, Morakot Kaewthamasorn^2^, Yudthana Samung^3^, Rangsan Jomtarak^4^, Kaung Myat Naing^5^, Teerawat Tongloy^5^, Santhad Chuwongin^5^ and Siridech Boonsang^6*^

^1^ _Faculty of Medicine, King Mongkut’s Institute of Technology Ladkrabang, Thailand_

^2^ _Veterinary Parasitology Research Unit, Faculty of Veterinary Science, Chulalongkorn University, Bangkok, Thailand_

^3^ _Faculty of Tropical Medicine, Mahidol University, Bangkok, Thailand_

^4^ _Faculty of Science and Technology, Suan Dusit University, Bangkok, Thailand._

^5^ _College of Advanced Manufacturing Innovation, King Mongkut’s Institute of Technology Ladkrabang, Thailand_

^6^ _Department of Electrical Engineering, School of Engineering, King Mongkut’s Institute of Technology Ladkrabang, Thailand_

*Corresponding author: [Siridech.Bo@kmitl.ac.th](mailto:Siridech.Bo@kmitl.ac.th)

**Supplementary Figures**


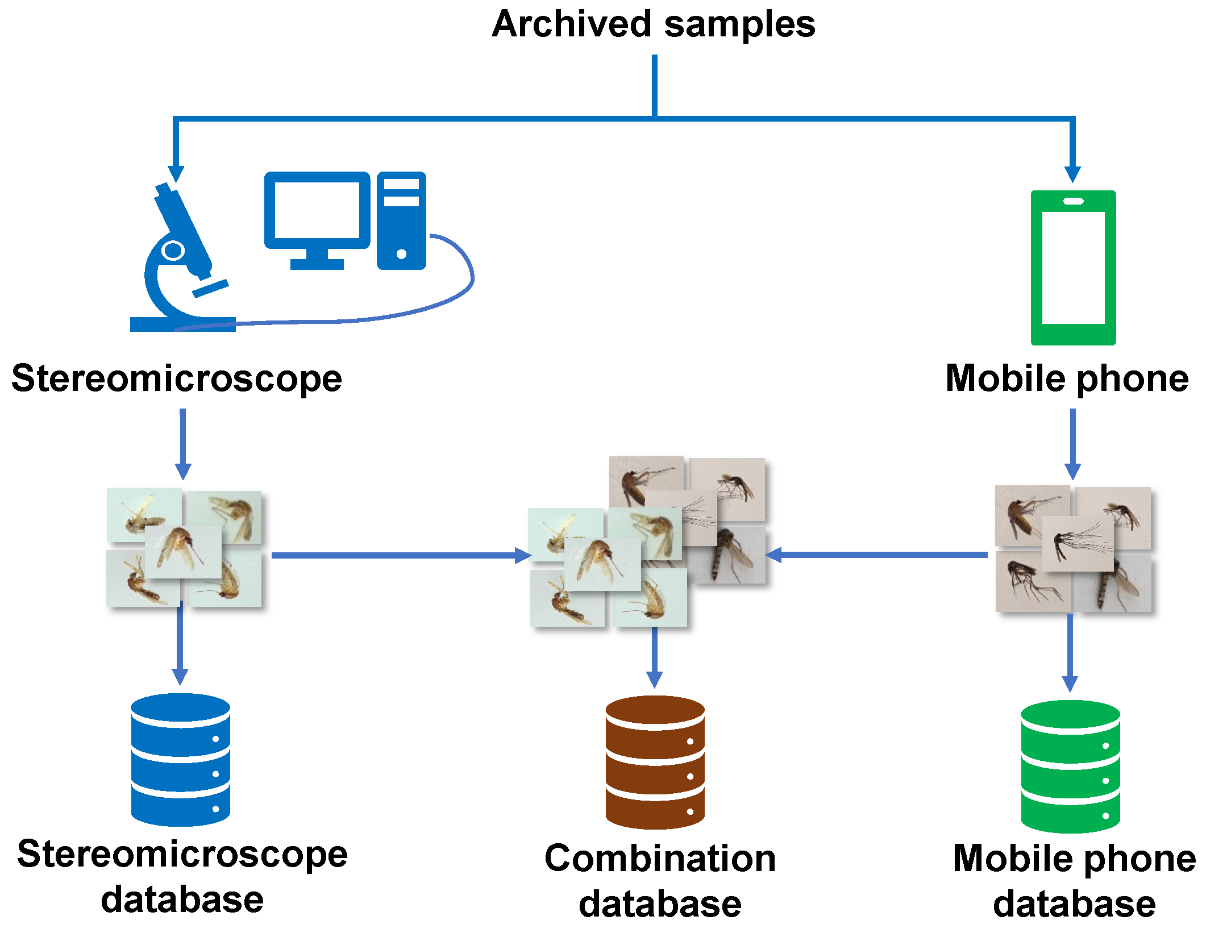


**Supplementary Figure S1. An illustration of the image capturing setup.** The experiment setup for images collections were composed of creating the stereomicroscope-, combination- and mobile phone databases, respectively. The figure was developed by using Microsoft PowerPoint 2013.


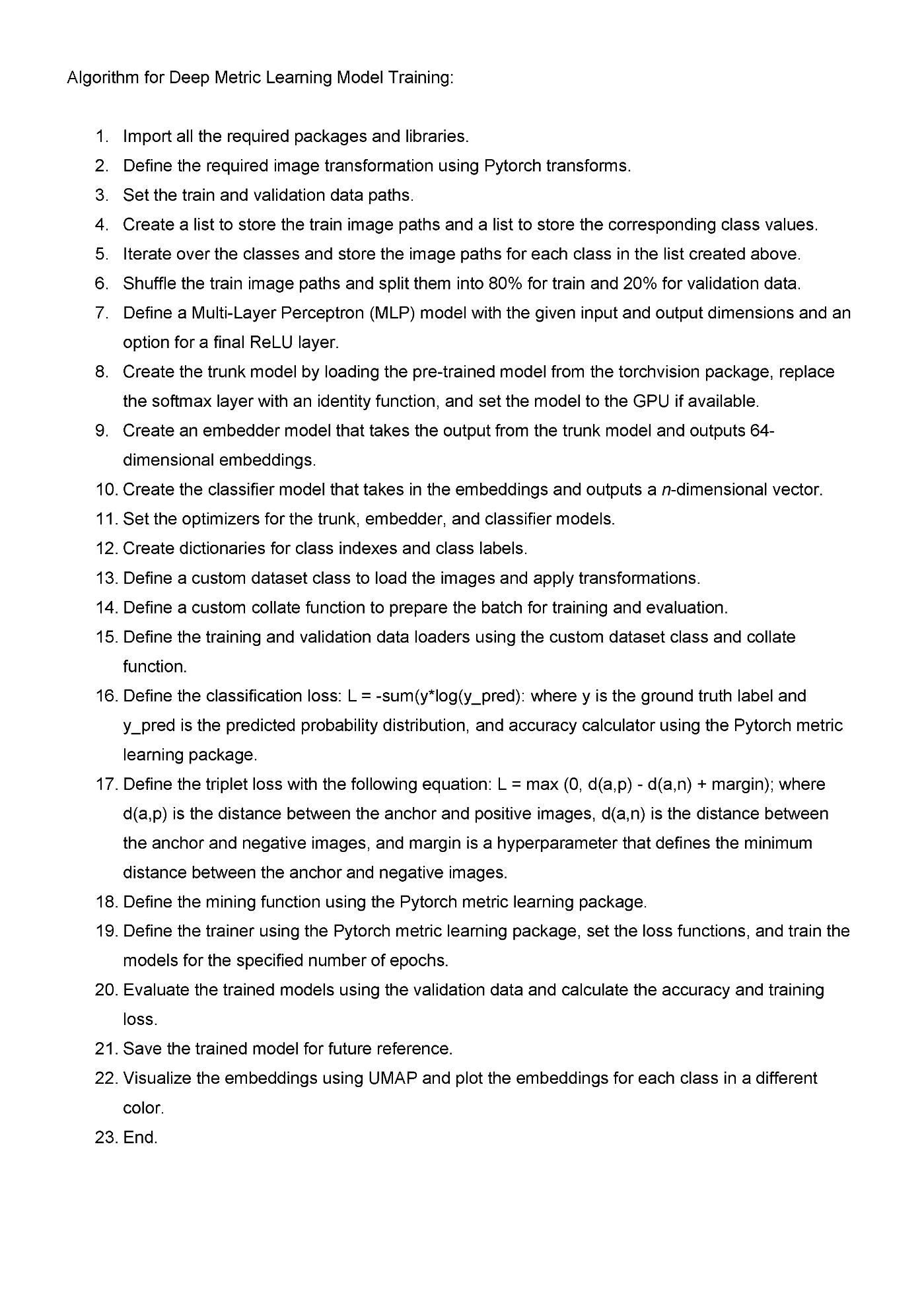


**Supplementary Figure S2. Explanation for the pseudocode of DML used.**


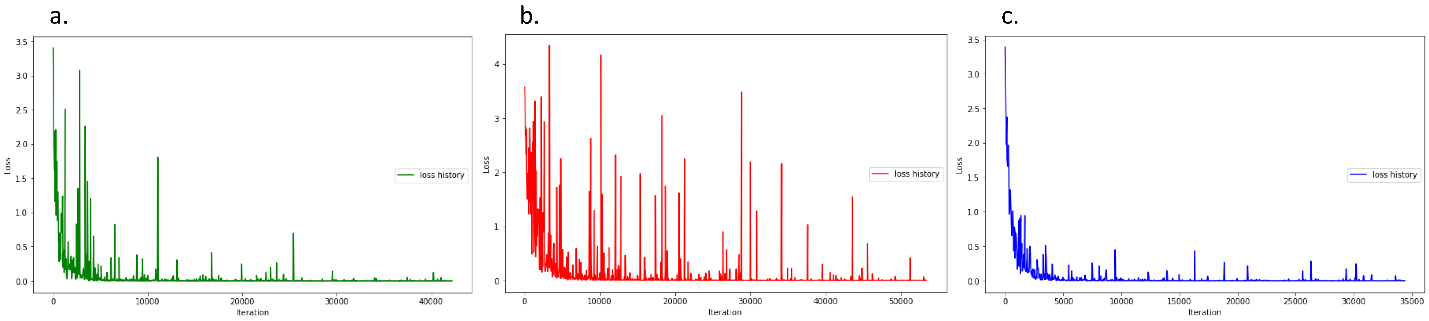


**Supplementary Figure S3. History of training loss per iteration.** The plot of Resnet-34 based models were trained with three different data including; a.) mobile phone dataset, b.) stereomicroscope dataset and c.) combination of both datasets mentioned as above, respectively.


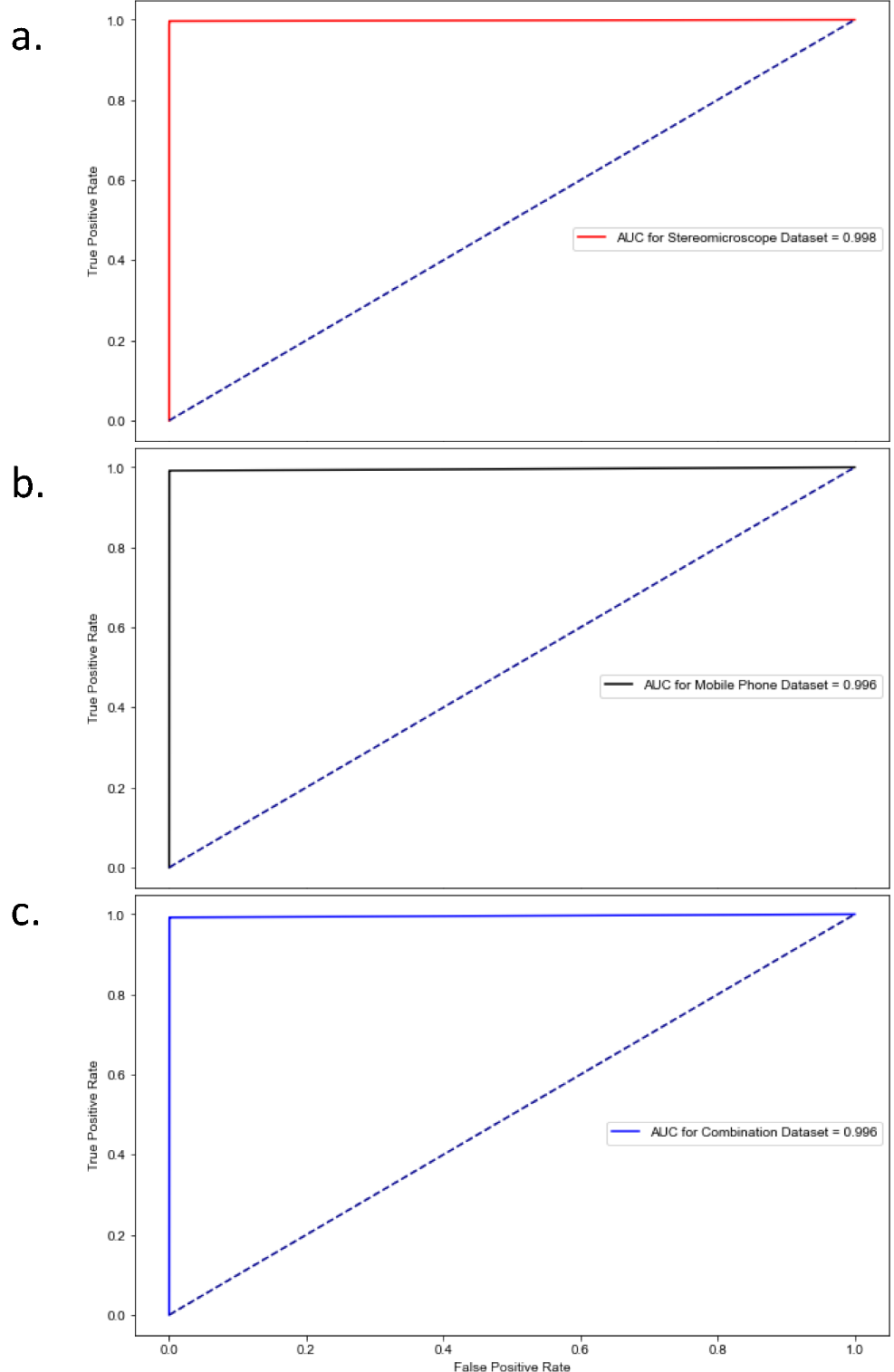


**Supplementary Figure S4. ROCs.** The proposed trained DML models were done with three datasets and showed; a.) the AUC of 0.998 for stereomicroscope, b.) the AUC of 0.996 for mobile phone and c.) the AUC for 0.996 for the combination datasets, respectively.


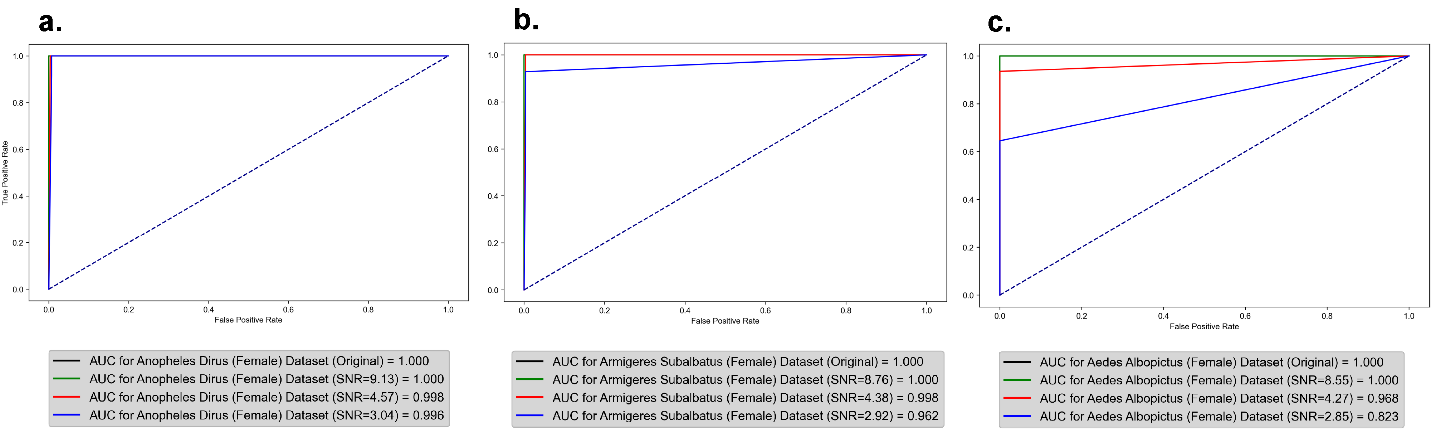


**Supplementary Figure S5. ROCs.** Three different degrees of Gaussian noise applied to images of three female mosquito species, namely *An. dirus* (a), *Ar. Subalbatus* (b) and *Ae. Albopictus* (c), respectively. The AUC under ROCs were analyzed to compare the model performance between an initial image to other three different levels of noise used.

**Supplementary Tables**

**Supplementary Table S1** Confusion matrix for testing dataset obtaining from the stereomicroscopic image source. The kNN used is 20 returned images from database.

|  | **Actual class** | | | | | | | | | | | | | | |
| --- | --- | --- | --- | --- | --- | --- | --- | --- | --- | --- | --- | --- | --- | --- | --- |
| **Predictive class** | Classes | sAdir_f | sAdir_m | sAeg_f | sAeg_m | sAlb_f | sAlb_m | sAsub_f | sCqui_f | sCqui_m | sCgel_f | sCvis_f | sMann_f | sMind_f | sMuni_f |
|  | sAdir_f | 12 | 0 | 0 | 0 | 0 | 0 | 0 | 0 | 0 | 0 | 0 | 0 | 0 | 0 |
|  | sAdir_m | 0 | 15 | 0 | 0 | 0 | 0 | 0 | 0 | 0 | 0 | 0 | 0 | 0 | 0 |
|  | sAeg_f | 0 | 0 | 14 | 0 | 0 | 0 | 0 | 0 | 0 | 0 | 0 | 0 | 0 | 0 |
|  | sAeg_m | 0 | 0 | 0 | 26 | 0 | 0 | 0 | 0 | 0 | 0 | 0 | 0 | 0 | 0 |
|  | sAlb_f | 0 | 0 | 0 | 0 | 31 | 0 | 0 | 0 | 0 | 0 | 0 | 0 | 0 | 0 |
|  | sAlb_m | 0 | 0 | 0 | 0 | 0 | 10 | 0 | 0 | 0 | 0 | 0 | 0 | 0 | 0 |
|  | sAsub_f | 0 | 0 | 0 | 0 | 0 | 0 | 28 | 0 | 0 | 0 | 0 | 0 | 0 | 0 |
|  | sCqui_f | 0 | 0 | 0 | 0 | 0 | 0 | 0 | 12 | 0 | 0 | 0 | 0 | 0 | 0 |
|  | sCqui_m | 0 | 0 | 0 | 0 | 0 | 0 | 0 | 0 | 15 | 0 | 0 | 0 | 0 | 0 |
|  | sCgel_f | 0 | 0 | 0 | 0 | 0 | 0 | 0 | 0 | 0 | 33 | 0 | 0 | 0 | 0 |
|  | sCvis_f | 0 | 0 | 0 | 0 | 0 | 0 | 0 | 0 | 0 | 1 | 23 | 0 | 0 | 0 |
|  | sMann_f | 0 | 0 | 0 | 0 | 0 | 0 | 0 | 0 | 0 | 0 | 0 | 15 | 0 | 0 |
|  | sMind_f | 0 | 0 | 0 | 0 | 0 | 0 | 0 | 0 | 0 | 0 | 0 | 0 | 44 | 0 |
|  | sMuni_f | 0 | 0 | 0 | 0 | 0 | 0 | 0 | 0 | 0 | 0 | 0 | 0 | 0 | 17 |

**Supplementary Table S2** Confusion matrix for testing dataset obtaining from the mobile phone image source. The kNN used is 20 returned images from database.

|  | **Actual class** | | | | | | | | | | |
| --- | --- | --- | --- | --- | --- | --- | --- | --- | --- | --- | --- |
| **Predictive class** | Classes | mNM | mAdir_f | mAdir_m | mAeg_f | mAeg_m | mAlb_f | mAlb_m | mAsub_f | mCqui_f | mCqui_m |
|  | mNM | 25 | 0 | 0 | 0 | 0 | 0 | 0 | 0 | 0 | 0 |
|  | mAdir_f | 0 | 8 | 0 | 0 | 0 | 0 | 0 | 0 | 0 | 0 |
|  | mAdir_m | 0 | 0 | 86 | 0 | 0 | 0 | 0 | 0 | 0 | 0 |
|  | mAeg_f | 0 | 1 | 0 | 17 | 0 | 0 | 1 | 0 | 0 | 0 |
|  | mAeg_m | 0 | 0 | 0 | 0 | 15 | 0 | 0 | 0 | 0 | 0 |
|  | mAlb_f | 0 | 0 | 0 | 0 | 0 | 113 | 0 | 0 | 0 | 0 |
|  | mAlb_m | 0 | 0 | 0 | 1 | 0 | 0 | 55 | 0 | 0 | 0 |
|  | mAsub_f | 0 | 0 | 0 | 0 | 0 | 0 | 0 | 62 | 0 | 0 |
|  | mCqui_f | 0 | 0 | 0 | 0 | 0 | 0 | 0 | 0 | 32 | 1 |
|  | mCqui_m | 0 | 0 | 0 | 0 | 0 | 0 | 0 | 0 | 0 | 55 |

**Supplementary Table S3** Confusion matrix for testing dataset obtaining from the combination of both image sources. The kNN used is 20 returned images from database.

|  | **Actual class** | | | | | | | | | | | | | | | |
| --- | --- | --- | --- | --- | --- | --- | --- | --- | --- | --- | --- | --- | --- | --- | --- | --- |
| **Predictive class** | Classes | mNM | Adir_f | Adir_m | Aeg_f | Aeg_m | Alb_f | Alb_m | Asub_f | Cqui_f | Cqui_m | Cgel_f | Cvis_f | Mann_f | Mind_f | Muni_f |
|  | mNM | 25 |  |  |  |  |  |  |  |  |  |  |  |  |  |  |
|  | Adir_f |  | 19 | 1 | 0 | 0 | 0 | 0 | 0 | 0 | 0 | 0 | 0 | 0 | 0 | 0 |
|  | Adir_m |  | 0 | 100 | 0 | 0 | 0 | 0 | 0 | 0 | 0 | 0 | 0 | 0 | 0 | 0 |
|  | Aeg_f |  | 1 | 0 | 31 | 0 | 0 | 0 | 0 | 0 | 0 | 0 | 0 | 0 | 0 | 0 |
|  | Aeg_m |  | 1 | 0 | 0 | 41 | 0 | 0 | 0 | 0 | 0 | 0 | 0 | 0 | 0 | 0 |
|  | Alb_f |  | 0 | 0 | 0 | 0 | 142 | 0 | 0 | 0 | 0 | 0 | 0 | 0 | 0 | 0 |
|  | Alb_m |  | 0 | 0 | 0 | 0 | 2 | 66 | 0 | 0 | 0 | 0 | 0 | 0 | 0 | 0 |
|  | Asub_f |  | 0 | 0 | 1 | 0 | 0 | 0 | 90 | 0 | 0 | 0 | 0 | 0 | 0 | 0 |
|  | Cqui_f |  | 0 | 0 | 0 | 0 | 0 | 0 | 0 | 44 | 0 | 0 | 0 | 0 | 0 | 0 |
|  | Cqui_m |  | 0 | 0 | 0 | 0 | 0 | 0 | 0 | 0 | 71 | 0 | 0 | 0 | 0 | 0 |
|  | Cgel_f |  | 0 | 0 | 0 | 0 | 0 | 0 | 0 | 0 | 0 | 34 | 0 | 0 | 0 | 0 |
|  | Cvis_f |  | 0 | 0 | 0 | 0 | 0 | 0 | 0 | 0 | 0 | 0 | 23 | 0 | 0 | 0 |
|  | Mann_f |  | 0 | 0 | 0 | 0 | 0 | 0 | 0 | 0 | 0 | 0 | 0 | 15 | 0 | 0 |
|  | Mind_f |  | 0 | 0 | 0 | 0 | 0 | 0 | 0 | 0 | 0 | 0 | 0 | 0 | 44 | 0 |
|  | Muni_f |  | 0 | 0 | 0 | 0 | 0 | 0 | 0 | 0 | 0 | 0 | 0 | 0 | 0 | 17 |

**Supplementary Table S4** Confusion matrix for a new combination testing-dataset obtaining from the first and second sources. The kNN used is 20 returned images from database.

|  | **Actual class** | | | | | | | | | |
| --- | --- | --- | --- | --- | --- | --- | --- | --- | --- | --- |
| **Predictive class** | Classes | Non-mosquito | Anopheles_f | Anopheles_m | Aedes_f | Aedes_m | Armigeres_f | Culex_f | Culex_m | Mansonia_f |
|  | Non-mosquito | 29 | 1 | 0 | 0 | 0 | 0 | 0 | 0 | 0 |
|  | Anopheles_f | 0 | 26 | 1 | 0 | 0 | 0 | 2 | 0 | 0 |
|  | Anopheles_m | 0 | 0 | 103 | 0 | 0 | 0 | 1 | 0 | 0 |
|  | Aedes_f | 0 | 1 | 0 | 181 | 1 | 2 | 3 | 0 | 0 |
|  | Aedes_m | 0 | 1 | 0 | 3 | 109 | 0 | 0 | 0 | 0 |
|  | Armigeres_f | 0 | 0 | 0 | 2 | 0 | 96 | 0 | 1 | 0 |
|  | Culex_f | 0 | 0 | 0 | 0 | 0 | 0 | 104 | 0 | 0 |
|  | Culex_m | 0 | 0 | 0 | 2 | 0 | 0 | 5 | 69 | 0 |
|  | Mansonia_f | 0 | 0 | 0 | 0 | 0 | 0 | 2 | 0 | 76 |

**Supplementary Table S5** Confusion matrix for a new stereomicroscope testing-dataset obtaining from the first and second sources. The kNN used is 20 returned images from database.

|  | **Actual class** | | | | | | | | | |
| --- | --- | --- | --- | --- | --- | --- | --- | --- | --- | --- |
| **Predictive class** | Classes | Non-mosquito | Anopheles_f | Anopheles_m | Aedes_f | Aedes_m | Armigeres_f | Culex_f | Culex_m | Mansonia_f |
|  | Non-mosquito | 29 | 0 | 0 | 0 | 0 | 0 | 0 | 0 | 0 |
|  | Anopheles_f | 0 | 16 | 0 | 0 | 0 | 0 | 3 | 0 | 0 |
|  | Anopheles_m | 0 | 0 | 18 | 0 | 0 | 0 | 0 | 1 | 0 |
|  | Aedes_f | 0 | 0 | 0 | 51 | 2 | 3 | 3 | 0 | 0 |
|  | Aedes_m | 0 | 0 | 0 | 2 | 37 | 0 | 0 | 0 | 0 |
|  | Armigeres_f | 0 | 0 | 0 | 0 | 0 | 29 | 0 | 1 | 0 |
|  | Culex_f | 0 | 0 | 0 | 0 | 0 | 0 | 70 | 2 | 0 |
|  | Culex_m | 0 | 0 | 0 | 1 | 0 | 0 | 0 | 17 | 0 |
|  | Mansonia_f | 0 | 0 | 0 | 0 | 0 | 0 | 2 | 0 | 76 |

**Supplementary Table S6** Confusion matrix for a new mobile phone testing-dataset obtaining from the first and second sources. The kNN used is 20 returned images from database.

|  | **Actual class** | | | | | | | | | |
| --- | --- | --- | --- | --- | --- | --- | --- | --- | --- | --- |
| **Predictive class** | Classes | Non-mosquito | Anopheles_f | Anopheles_m | Aedes_f | Aedes_m | Armigeres_f | Culex_f | Culex_m | Mansonia_f |
|  | Non-mosquito | 29 | 1 | 0 | 0 | 0 | 0 | 0 | 0 | 0 |
|  | Anopheles_f | 0 | 10 | 1 | 0 | 0 | 0 | 0 | 0 | 0 |
|  | Anopheles_m | 0 | 0 | 85 | 0 | 0 | 0 | 1 | 0 | 0 |
|  | Aedes_f | 0 | 1 | 0 | 130 | 0 | 0 | 0 | 0 | 0 |
|  | Aedes_m | 0 | 1 | 0 | 1 | 71 | 0 | 0 | 0 | 0 |
|  | Armigeres_f | 0 | 0 | 0 | 2 | 0 | 66 | 0 | 1 | 0 |
|  | Culex_f | 0 | 0 | 0 | 0 | 0 | 0 | 33 | 0 | 0 |
|  | Culex_m | 0 | 0 | 0 | 1 | 0 | 0 | 5 | 54 | 0 |
|  | Mansonia_f | 0 | 0 | 0 | 0 | 0 | 0 | 0 | 0 | 76 |

**Supplementary Table S7.** Performance comparison of the mobile phone dataset trained Resnet-34-DML with (KNN=20)/ without the CBIR (K=1) system. Bold numbers are representative for highest values.

| **No.** | **Species** | **Accuracy** | | **Specificity** | | **Precision** | | **Recall** | | **F1 score** | |
| --- | --- | --- | --- | --- | --- | --- | --- | --- | --- | --- | --- |
|  |  | **k = 1** | **k = 20** | **k = 1** | **k = 20** | **k = 1** | **k = 20** | **k = 1** | **k = 20** | **k = 1** | **k = 20** |
| 1 | Non-mosquito (*Musca domestica*) | 1.000 | 1.000 | 1.000 | 1.000 | 1.000 | 1.000 | 1.000 | 1.000 | 1.000 | 1.000 |
| 2 | *Anopheles dirus*, female | 0.998 | 0.998 | 1.000 | 1.000 | 1.000 | 1.000 | 0.889 | 0.889 | 0.941 | 0.941 |
| 3 | *Anopheles dirus*, male | 1.000 | 1.000 | 1.000 | 1.000 | 1.000 | 1.000 | 1.000 | 1.000 | 1.000 | 1.000 |
| 4 | *Aedes aegypti*, female | **0.996** | 0.994 | **0.998** | 0.996 | **0.944** | 0.895 | 0.944 | 0.944 | **0.944** | 0.919 |
| 5 | *Aedes aegypti*, male | 0.998 | **1.000** | 1.000 | 1.000 | 1.000 | 1.000 | 0.933 | **1.000** | 0.966 | **1.000** |
| 6 | *Aedes albopictus*, female | 1.000 | 1.000 | 1.000 | 1.000 | 1.000 | 1.000 | 1.000 | 1.000 | 1.000 | 1.000 |
| 7 | *Aedes albopictus*, male | 0.996 | 0.996 | 0.995 | **0.998** | 0.966 | **0.982** | **1.000** | 0.982 | 0.982 | 0.982 |
| 8 | *Armigeres subalbatus*, female | 1.000 | 1.000 | 1.000 | 1.000 | 1.000 | 1.000 | 1.000 | 1.000 | 1.000 | 1.000 |
| 9 | *Culex quinquefasciatus*, female | **1.000** | 0.998 | **1.000** | 0.998 | **1.000** | 0.970 | 1.000 | 1.000 | **1.000** | 0.985 |
| 10 | *Culex quinquefasciatus*, male | **1.000** | 0.998 | 1.000 | 1.000 | 1.000 | 1.000 | **1.000** | 0.982 | **1.000** | 0.991 |
|  | **Average** | ***0.999*** | *0.998* | *0.999* | *0.999* | ***0.991*** | *0.985* | *0.977* | ***0.980*** | ***0.983*** | *0.982* |

**Supplementary Table S8.** Performance comparison of the stereomicroscope dataset trained Resnet-34-DML with (kNN=20)/ without the CBIR (k=1) system. Bold numbers are representative for highest values.

| **No.** | **Species** | **Accuracy** | | **Specificity** | | **Precision** | | **Recall** | | **F1 score** | |
| --- | --- | --- | --- | --- | --- | --- | --- | --- | --- | --- | --- |
|  |  | **k = 1** | **k = 20** | **k = 1** | **k = 20** | **k = 1** | **k = 20** | **k = 1** | **k = 20** | **k = 1** | **k = 20** |
| 1 | *Anopheles dirus*, female | 1.000 | 1.000 | 1.000 | 1.000 | 1.000 | 1.000 | 1.000 | 1.000 | 1.000 | 1.000 |
| 2 | *Anopheles dirus*, male | 1.000 | 1.000 | 1.000 | 1.000 | 1.000 | 1.000 | 1.000 | 1.000 | 1.000 | 1.000 |
| 3 | *Aedes aegypti*, female | 0.997 | **1.000** | 0.996 | **1.000** | 0.933 | **1.000** | 1.000 | 1.000 | 0.966 | **1.000** |
| 4 | *Aedes aegypti*, male | 0.997 | **1.000** | 1.000 | 1.000 | 1.000 | 1.000 | 0.962 | **1.000** | 0.980 | **1.000** |
| 5 | *Aedes albopictus*, female | 1.000 | 1.000 | 1.000 | 1.000 | 1.000 | 1.000 | 1.000 | 1.000 | 1.000 | 1.000 |
| 6 | *Aedes albopictus*, male | 1.000 | 1.000 | 1.000 | 1.000 | 1.000 | 1.000 | 1.000 | 1.000 | 1.000 | 1.000 |
| 7 | *Armigeres subalbatus, female* | 1.000 | 1.000 | 1.000 | 1.000 | 1.000 | 1.000 | 1.000 | 1.000 | 1.000 | 1.000 |
| 8 | *Culex quinquefasciatus*, female | 1.000 | 1.000 | 1.000 | 1.000 | 1.000 | 1.000 | 1.000 | 1.000 | 1.000 | 1.000 |
| 9 | *Culex quinquefasciatus*, male | 1.000 | 1.000 | 1.000 | 1.000 | 1.000 | 1.000 | 1.000 | 1.000 | 1.000 | 1.000 |
| 10 | *Culex gelidus*, female | 0.997 | 0.997 | 1.000 | 1.000 | 1.000 | 1.000 | 0.971 | 0.971 | 0.985 | 0.985 |
| 11 | *Culex vishnui*, female | 0.997 | 0.997 | 0.996 | 0.996 | 0.958 | 0.958 | 1.000 | 1.000 | 0.979 | 0.979 |
| 12 | *Mansonia annularis*, female | 1.000 | 1.000 | 1.000 | 1.000 | 1.000 | 1.000 | 1.000 | 1.000 | 1.000 | 1.000 |
| 13 | *Mansonia Indiana*, female | 1.000 | 1.000 | 1.000 | 1.000 | 1.000 | 1.000 | 1.000 | 1.000 | 1.000 | 1.000 |
| 14 | *Mansonia uniformis*, female | 1.000 | 1.000 | 1.000 | 1.000 | 1.000 | 1.000 | 1.000 | 1.000 | 1.000 | 1.000 |
|  | **Average** | *0.999* | ***1.000*** | *0.999* | ***1.000*** | *0.992* | ***0.997*** | *0.995* | ***0.998*** | *0.994* | ***0.997*** |

**Supplementary Table S9.** Performance comparison of the combination dataset trained Resnet-34-DML with (kNN=20)/ without the CBIR (k=1) system. Bold numbers are representative for highest values.

| **No.** | **Species** | **Accuracy** | | **Specificity** | | **Precision** | | **Recall** | | **F1 score** | |
| --- | --- | --- | --- | --- | --- | --- | --- | --- | --- | --- | --- |
|  |  | **k = 1** | **k = 20** | **k = 1** | **k = 20** | **k = 1** | **k = 20** | **k = 1** | **k = 20** | **k = 1** | **k = 20** |
| 1 | Non-mosquito (*Musca domestica*) | 1.000 | 1.000 | 1.000 | 1.000 | 1.000 | 1.000 | 1.000 | 1.000 | 1.000 | 1.000 |
| 2 | *Anopheles dirus*, female | **0.997** | 0.996 | 1.000 | 0.999 | **1.000** | 0.950 | 0.905 | 0.905 | **0.950** | 0.927 |
| 3 | *Anopheles dirus*, male | **1.000** | 0.999 | 1.000 | 1.000 | 1.000 | 1.000 | **1.000** | 0.990 | **1.000** | 0.995 |
| 4 | *Aedes aegypti*, female | 0.997 | 0.997 | 0.999 | 0.999 | 0.969 | 0.969 | 0.969 | 0.969 | 0.969 | 0.969 |
| 5 | *Aedes aegypti*, male | 0.999 | 0.999 | 0.999 | 0.999 | 0.976 | 0.976 | 1.000 | 1.000 | 0.988 | 0.988 |
| 6 | *Aedes albopictus*, female | 0.996 | **0.997** | 1.000 | 1.000 | 1.000 | 1.000 | 0.979 | **0.986** | 0.989 | **0.993** |
| 7 | *Aedes albopictus*, male | 0.996 | 0**.997** | 0.996 | 0**.997** | 0.957 | **0.971** | 1.000 | 1.000 | 0.978 | **0.985** |
| 8 | *Armigeres subalbatus*, female | 0.999 | 0.999 | 0.999 | 0.999 | 0.989 | 0.989 | 1.000 | 1.000 | 0.994 | 0.994 |
| 9 | *Culex quinquefasciatus*, female | 1.000 | 1.000 | 1.000 | 1.000 | 1.000 | 1.000 | 1.000 | 1.000 | 1.000 | 1.000 |
| 10 | *Culex quinquefasciatus*, male | 1.000 | 1.000 | 1.000 | 1.000 | 1.000 | 1.000 | 1.000 | 1.000 | 1.000 | 1.000 |
| 11 | *Culex gelidus*, female | 1.000 | 1.000 | 1.000 | 1.000 | 1.000 | 1.000 | 1.000 | 1.000 | 1.000 | 1.000 |
| 12 | *Culex vishnui*, female | 1.000 | 1.000 | 1.000 | 1.000 | 1.000 | 1.000 | 1.000 | 1.000 | 1.000 | 1.000 |
| 13 | *Mansonia annularis*, female | 1.000 | 1.000 | 1.000 | 1.000 | 1.000 | 1.000 | 1.000 | 1.000 | 1.000 | 1.000 |
| 14 | *Mansonia Indiana*, female | 1.000 | 1.000 | 1.000 | 1.000 | 1.000 | 1.000 | 1.000 | 1.000 | 1.000 | 1.000 |
| 15 | *Mansonia uniformis*, female | 1.000 | 1.000 | 1.000 | 1.000 | 1.000 | 1.000 | 1.000 | 1.000 | 1.000 | 1.000 |
|  | **Average** | *0.999* | *0.999* | *0.999* | *0.999* | ***0.993*** | *0.990* | *0.990* | *0.990* | ***0.991*** | *0.990* |
